# The Representation of Coordinate Relations in Lexical Semantic Memory

**DOI:** 10.3389/fpsyg.2020.00098

**Published:** 2020-02-11

**Authors:** Thomas M. Gruenenfelder

**Affiliations:** Department of Psychological and Brain Sciences, Indiana University Bloomington, Bloomington, IN, United States

**Keywords:** coordinate relations, semantic memory, lexical semantic memory, semantic networks, associative networks, co-hyponyms, hyponyms, semantic relations

## Abstract

Two experiments examined the size of the typicality effect for true items in a category verification task as a function of the type of false item used. In Experiment 1, compared to the case where false items paired unrelated concepts (“carrot–vehicle”), the typicality effect was much larger when false items paired an exemplar with a category coordinate to its proper category (“carrot–fruit”). In contrast, when false items paired coordinate concepts (“carrot–pea”) or reversed the ordering of subject and predicate terms (“All vegetables are carrots”), the typicality effect did not change in size. Further, the time to verify true sentences did not increase monotonically with the semantic similarity of the two terms used in false sentences. Experiment 2 showed that the pattern of results for coordinate items reflected semantic processing, not simply task difficulty. A combined analysis examined data across multiple experiments, increasing the power of the statistical analysis. The size of the typicality effect when coordinate false items were used was again the same as when false items paired unrelated concepts. The most straightforward explanation of this pattern of results seems to be in terms of a *sparse semantic network* model of lexical semantic memory, in which labeled links are used to indicate the semantic relation that exists between pairs of words.

## Introduction

Theories of lexical semantic memory are concerned with how people represent the meanings of words in the mind, and the processes that operate on those meanings. The purpose of the present paper is to replicate and extend an important empirical result from the literature on tests of such theories, and to discuss the theoretical significance of that result. That empirical result concerns how the size of the typicality effect for true stimuli in a category verification task changes as a function of the semantic relation between the concepts used in false stimuli. The theoretical implications concern how those relations are represented in memory, and in particular which relations are best captured by associative network models (e.g., Collins and Quillan, 1969; Glass and Holyoak, 1975; Holyoak and Glass, 1975) and which are best captured by feature models (Rips et al., 1973; Smith et al., 1974a, b; Gellatly and Gregg, 1975, 1977; McCloskey and Glucksberg, 1979; Moss et al., 1994; Masson, 1995; McRae et al., 1997; Pexman et al., 2002, 2003; Hino et al., 2006) or distributional models (e.g., Lund and Burgess, 1996; Landauer and Dumais, 1997; Burgess, 1998; Jones and Mewhort, 2007; Mikolov et al., 2013).

In a category verification task, participants are asked to verify as quickly as possible sentences of the form “A *subject* is a *predicate*,” (“Peas are vegetables” is an example of a true stimulus). Participants are usually assumed to know the correct answer, and the dependent variable of most interest is their time to respond. The typicality effect refers to the finding that more typical category exemplars (sparrow–bird) are verified to be members of a category more quickly than less typical members (hawk–bird) (e.g., Rips et al., 1973; Rosch, 1975).

Several experiments have examined how the size of the typicality effect for true stimuli changes as a function of the semantic relation between the two terms in false stimuli. In their Experiment 2, McCloskey and Glucksberg (1979) found a larger typicality effect in what is termed here a *Cross-Category* condition than in what is termed here an *Anomalous* condition. In a Cross-Category condition, false items pair exemplars with a category coordinate to its proper category (e.g., pear–vegetable). In an Anomalous condition, false items pair semantically unrelated terms (e.g., pear–vehicle). Throughout this paper, the size of the typicality effect in the Anomalous condition is used as a baseline for comparing its size in other conditions.

McCloskey and Glucksberg (1979) used *Reversed* false items in their first experiment and found no change in the size of the typicality effect (again, in comparison to its size in an Anomalous condition). In a Reversed false stimulus, the role of the subject and predicate terms of a true stimulus are reversed (“All pears are fruits” becomes “All fruits are pears”). Similarly, in his Experiment 1, Gruenenfelder (1986) observed the same size typicality effect in two *Coordinate* conditions as in an Anomalous condition. In a Coordinate condition, false items pair coordinate terms, i.e., two exemplars from the same category that can be used to contrast with one another, i.e., are mutually exclusive (e.g., pear–peach) and usually share an immediate superordinate.

These three studies are the only ones that I am aware of that provide for a non-confounded examination of how the typicality effect varies as a function of the type of semantic relation used in the false stimuli.

How can various approaches to modeling lexical semantic memory explain these context effects, i.e., changes or lack thereof in the size of the typicality effect as a function of the type of semantic relation expressed in false stimuli? Feature models (Rips et al., 1973; Smith et al., 1974b; Gellatly and Gregg, 1975, 1977; McCloskey and Glucksberg, 1979; Moss et al., 1994; Masson, 1995; McRae et al., 1997, 2005; Pexman et al., 2002, 2003; Hino et al., 2006) represent a word’s meaning as a list of features or properties possessed by the concept represented by that word. In these models, category verification has been hypothesized to involve a comparison of the features or attributes of the subject term to those of the predicate term. A “true” decision is made when a criterion number of matching features is found. Typical exemplars presumably share more features with the category than do atypical exemplars. Hence, matches accumulate at a faster rate for more typical exemplars, resulting in the typicality effect. Similarly, a “false” decision is made when a criterion number of mismatching features is found. This process essentially computes the semantic similarity of the two terms in a stimulus. As such, it is likely to fail when false items pair highly similar terms. Nevertheless, the approach is discussed in some detail here because it is highly developed, because it may work quite well to explain some context effects, and because it has been the subject of extensive earlier empirical work.

Context effects, where reaction times increase in the presence of false items pairing semantically similar terms (Relative to Anomalous false items, the Coordinate, Cross-Category, and Reversed false items all pair semantically similar terms.), have been explained by feature comparison models by assuming an increase in the criteria number of matching features that must be found in order to decide that the stimulus is true (e.g., Gellatly and Gregg, 1975, 1977; McCloskey and Glucksberg, 1979; Hampton, 1997). Because more matching features must be found when false items pair semantically related terms than when the false items are Anomalous, overall reaction times increase. Because feature matches accumulate more slowly for atypical than typical exemplars, the resulting increase in reaction times is larger for atypical than typical exemplars. That is, the typicality effect increases. The mechanics of the decision process can be modeled in a variety of ways: as independent counters accumulating evidence for true and false decisions (Gellatly and Gregg, 1975, 1977), as a Bayesian decision rule (McCloskey and Glucksberg, 1979) or as a random walk (Link, 1975) or diffusion process (Ratcliff, 1978; Ratcliff and Rouder, 1998). Under reasonable assumptions, all these variants make the same general prediction of a larger increase in reaction time to atypical than typical exemplars when false items pair semantically similar terms. This prediction of an increased typicality effect is in accord with the findings in the Cross-Category condition of McCloskey and Glucksberg’s Experiment 2, but not with the Reversed condition of their Experiment 1 nor the Coordinate conditions of Gruenenfelder’s (1986) Experiment 1. In those two conditions, the typicality effect did not change. Hence, if the finding of equal-sized typicality effects across the Anomalous, Coordinate, and Reversed conditions is confirmed, it would challenge this particular explanation of context effects (i.e., of an increase in the criteria number of matching features that must be found in order to reach a “true” decision) in these conditions.

Distributional or high dimensional spatial models of semantic memory represent the meaning of a concept as a point in a high-dimensional space (e.g., Lund and Burgess, 1996; Landauer and Dumais, 1997; Burgess, 1998; Jones and Mewhort, 2007; Mikolov et al., 2013). That point can be described as a vector where each element of the vector is the value that concept possesses on the corresponding dimension of the space. For reasons discussed in the Supplementary Material, in the context of the present experiments, the dimensions are treated as features, and hence the models make predictions similar to those of more traditional feature models.

The difficulty feature and distributional models, as formulated above, have explaining the invariance of the typicality effect across the Anomalous, Reversed, and Coordinate conditions is perhaps not surprising. They model category verification as a similarity judgment task. Such a strategy could well work in the Cross-Category condition. There is presumably a gradient in the number of shared features between the two terms in a false stimulus, with that number being lowest for the Anomalous items, higher for Cross-Category false stimuli, higher still for atypical true stimuli, and highest for typical true stimuli. Hence, the number of shared features could potentially discriminate true from false stimuli in the Cross-Category condition. When the semantic similarity of the terms in false items is very high, however, such as is the case in the Reversed and Coordinate conditions, and overlaps with the semantic similarity of the terms in true items, judgments of similarity simply do not reliably discriminate true from false items. How then might these models approach category verification in a context of Reversed and Coordinate false items?

One possibility, at least for the Coordinate condition, is that participants focus on distinguishing features, which have been shown to be particularly salient (Cree et al., 2006). Two comments on such a proposal are in order. First, it is not obvious under this hypothesis why the typicality effect would be the same size in the Coordinate condition as in the Anomalous condition, where, according to feature models, an overall similarity judgment presumably distinguishes false from true stimuli. Does the qualitatively different strategy in the Coordinate condition of directing attention toward distinguishing features just coincidentally result in a typicality effect that is the same size as in the Anomalous condition? Second, features are not distinguishing in and of themselves; they are only distinguishing in terms of how they relate two concepts. The feature *barks* perhaps distinguishes the concept *dog* from *cat*, *cow*, and *horse*, but not from *coyote*, *hyena*, or *seal*. Further, the fact that two concepts differ on a feature does not make them coordinates. *Birds* fly; *penguins* do not, but *bird* and *penguin* are not coordinate concepts. The feature needs to be marked as *distinguishing* as it relates to two specific concepts. This hypothesis seems very similar to a semantic network model (see below), i.e., to claiming that an association exists between the two concepts and the association is labeled as indicating a coordinate relation, albeit perhaps with additional information about which features distinguish the two concepts.

A generalization of that second approach may be more promising. Perhaps different features are involved in a feature comparison process depending upon the particular semantic discrimination required in a particular condition^1^ (e.g., Baroni et al., 2012; Lenci and Benotto, 2012; Roller et al., 2014; Weeds et al., 2014; Nguyen et al., 2016, 2017a; Shwartz et al., 2016). More specifically, in the Anomalous condition, as is the case for more traditional feature comparison theories, an overall similarity comparison is sufficient to distinguish true from false stimuli. In the Cross-Category condition, the same process is used but with a higher criterion for the number of matching features that must be found to support a true decision, resulting in longer overall reaction times and a larger typicality effect. In the Reversed condition a different set of features, capable of discriminating the direction of a set–superset relation is examined. And in the Coordinate condition, a third set of features is examined, capable of discriminating set–superset relations from coordinate relations. What is not clear in this approach, and what seems to rely on a coincidence, is why, given that different sets of features are used in the three conditions, the typicality effect is the same size across the Anomalous, Coordinate, and Reversed conditions^2^. This approach is consistent with a typicality effect that changes in the Cross-Category condition relative to the Anomalous condition, but seems to have more difficulty explaining equal size typicality effects across the Anomalous, Coordinate, and Reversed conditions, where presumably different feature sets are being used to determine the semantic relation.

Semantic network models of lexical semantic memory make a quite different prediction. In semantic network models, words (or the concepts that they denote) are linked together in a network via labeled associations, where the label indicates the semantic relation between the two words (Collins and Quillan, 1969; Glass and Holyoak, 1975; Holyoak and Glass, 1975)^3^. Early network models focused on subset-superset, or *isa*, links, where isa (car, vehicle) indicates that a car is a vehicle, and property links, such as has (car, wheels), indicating that a car has wheels.

Of particular concern here are the associations from an exemplar to a category term (denoted here as *SUBSET* relations) or vice versa (*SUPERSET* relations) and associations between two coordinate terms, i.e., two words from the same category and that are used to contrast with one another (abbreviated as *COORD* below). The network is sparse in the sense that some category exemplars, in particular some atypical exemplars, may not necessarily have a direct link to the category term. *Chicken*, for example, may not be directly associated to *bird*, but only indirectly, through another exemplar of bird, such as *hawk*: *chicken*–*hawk*–*bird*, or more explicitly, COORD (chicken, hawk), SUBSET (hawk, bird). Note that retrieval of such a path would be sufficient in a category verification task to conclude that chickens are in fact birds in a context of, for instance, coordinate false items but not in a context of cross-category false items. That is, in some contexts, we can infer that a chicken is a bird, because chicken is a coordinate of hawk, and hawks are birds. Similarly, not all exemplars of a given category would necessarily be linked by a coordinate relation–it all depends on the person’s previous learning experiences. Retrieval of a chain of two coordinate relations–COORD (chicken, hawk), COORD (hawk, eagle)–would consistently indicate a coordinate relation between the first and last terms (chicken, eagle), regardless of the context. Finally, there is nothing to prevent an exemplar from forming a subset or superset relation with a category coordinate to its proper category, nor is there anything to prevent two exemplars from different categories from forming a coordinate relation, again dependent upon the person’s learning experiences. *Apple*, for example, may form a subset relation with *vegetable* as well as with *fruit*. Likewise, *apple* may form coordinate relations not only with *kumquat* and *strawberry*, but also with *carrot* and *beans*.

Verification of a stimulus in the category verification task is assumed to follow a two-stage process. The first stage involves an attempt to retrieve an association, or a chain of associations, between the subject and predicate terms of the stimulus. Two retrieval processes are assumed to be carried out in parallel, one beginning from the subject term, the other from the predicate term. Retrieval in the network consists of traversing edges or links first from the node (word) from which the retrieval process is initiated, then traversing edges from those retrieved nodes to additional nodes to which they are directly connected, then traversing edges from those additionally retrieved nodes to the nodes to which they are directly connected, and so on. People are assumed to be able to choose which edges to traverse based on the label on that edge, i.e., the semantic relation it represents. In the category verification task, only edges with SUBSET, SUPERSET, and COORD labels are assumed to be traversed. The process terminates when either an association or chain of associations linking the two terms and sufficient for making the required discrimination is retrieved, or when all paths of associations less than or equal to a particular *stopping length* are retrieved without retrieving a chain linking the two terms. In that latter event–a failure to retrieve a chain of associations linking the subject and predicate terms–the assumption is that it is relatively safe to conclude that the two terms are not semantically related, i.e., that the stimulus is an Anomalous false (cf. King and Anderson, 1976).

Associations vary in strength, presumably reflecting the frequency with which the two words co-occur with one another relative to their total frequency of occurrence (Jaccard, 1912; Church and Hanks, 1990). Stronger associations are retrieved more quickly than weaker associations. More precisely, and following Lorch (1982), stronger associations are selected for additional processing earlier than weaker associations. In the context of a category verification task, that additional processing consists of determining, first, whether the retrieved concept matches the second word in the stimulus. If so, the retrieval process can end. If not, and if the stopping length has not been reached, retrieval of that path continues from the just retrieved concept. If not, and the stopping length has been reached, retrieval of that path is terminated (and the path can be discarded). In some experimental conditions (see below), in the case where a path is retrieved, that additional processing may also involve using the label on the retrieved association (cf. Chaffin and Herrmann, 1984; Gruenenfelder, 1986), as described below.

Depending upon the discrimination required between true and false stimuli, the retrieval stage may be sufficient to make a response. In particular, in the Anomalous condition, where all false stimuli are Anomalous, if an associative path is retrieved between the subject and predicate terms, a true response is made. If no such path can be retrieved within the stopping length, a false response is made. Typical exemplars presumably are more strongly associated with the category term than atypical exemplars, resulting in faster retrieval for typical exemplars, and hence a typicality effect. In addition, atypical exemplars are more likely to require that a chain of associations be retrieved in order to find the category term. For example, to determine that okra is a vegetable, it may first be necessary to retrieve a coordinate relation between okra and beans and then a set–superset relation from beans to vegetables. The need to retrieve a second relation would also contribute to the typicality effect.

When false stimuli pair semantically related concepts, a second stage of processing, referred to here as *path evaluation*, is necessary in order to discriminate true from false stimuli. This process is necessary in both the Coordinate and Reversed conditions since it is possible to retrieve a path of associations between the subject and predicate terms for both true and false stimuli. It would also be necessary in the Cross-Category condition, to the extent that the network is even capable of discriminating true and false stimuli in this condition (see the section “General Discussion”). The path evaluation process involves examining the labels on the retrieved associations linking the subject and predicate terms in order to determine whether the stimulus represents a set–superset relation or, in the Coordinate condition, a coordinate relation or, in the Reversed condition, a superset–set relation. That process is described in more detail in the section “General Discussion.”

The path evaluation process is responsible for the overall increase in reaction times in the Coordinate and Reversed conditions. That evaluation process, however, is not affected by associative strength, and hence typicality does not affect the duration of the evaluation process (cf. Gruenenfelder, 1986). Access to the properties of the retrieved association, such as the second concept linked to by the first, is one definition of what it means to retrieve an association (Chaffin and Herrmann, 1981, 1984; Lorch, 1982). It may take longer to gain access to that information for an atypical exemplar (i.e., to retrieve the association), but once access is gained, the information can be processed equally efficiently for atypical as for typical exemplars. Consequently, the duration of the path evaluation process and hence the increase in reaction times is the same for typical and atypical exemplars in the conditions pairing semantically related items as in the Anomalous condition. Hence, the typicality effect is the same size in the Coordinate and Reversed conditions as in the Anomalous condition.

As reviewed above, the empirical results to date indicate that relative to an Anomalous condition, the typicality effect does not change in size in the Coordinate or Reversed conditions but does increase in the Cross-Category condition. That pattern of results would suggest that a semantic network is used to discriminate true from false stimuli in the Anomalous, Coordinate, and Reversed conditions, but that some other process–perhaps a similarity judgment–is used in the Cross-Category condition. For several reasons, those results need to be replicated. First, despite their potential theoretical significance, there is only one experiment concerning each of the three different types of false stimuli (Cross-Category, Reversed, and Coordinate). Second, the findings for Reversed false items are based on a relatively small number of participants (8), making it difficult to accept the null hypothesis of no change. Third, the three studies did not all use the same true items. Hence, it is unknown whether the different results in the Cross-Category condition compared to the Reversed and Coordinate conditions reflect different effects of the different types of false stimuli, or simply differences in the true items used. Accordingly, Experiment 1 examined the relative size of the typicality effect for true items in different false conditions, where the false conditions differed from one another in terms of the type of false stimuli used: Anomalous, Cross-Category, Reversed, or Coordinate false items. Across participants, the same true items were used in all four conditions.

## Experiment 1

Experiment 1 examined the size of the typicality effect for true stimuli in a category verification task as a function of the type of relation exhibited by false stimuli. Each participant served in four conditions. In the Anomalous condition, all false items paired semantically unrelated concepts (pea–furniture); this condition served as the baseline against which others were compared. In the Coordinate condition, all false items paired coordinate items (pea–bean). In the Cross-Category condition, all false items paired an exemplar with a concept coordinate to its proper category (pea–fruit). Finally, in the Reversed condition, false items reversed the order of the subject and predicate terms of a true item (vegetable–pea).

Although no participant saw any true stimulus more than once, across participants the same true items were used in all four conditions. Hence, any differences in the size of the typicality effect across the conditions can be attributed to the type of false stimuli used in those conditions. Changes in the size of the typicality effect in the three conditions using semantically similar false items (Coordinate, Cross-Category, and Reversed), relative to its size in the Anomalous condition, can be used to make inferences concerning the mental representations used to make the required semantic discriminations in the various conditions.

To determine the effect of a type of false stimulus on response times to true stimuli, participants must in some sense know what type of false stimulus is used in each condition. For that reason, the type of false stimulus used was described in the instructions that immediately preceded participation in that condition. In addition, each condition began with 20 practice trials in order to familiarize participants with the type of false stimulus used in that condition.

### Method

#### Participants

Twenty-four native speakers of English participated in the experiment in partial fulfillment of an Indiana University introductory psychology course requirement. The Indiana University Institutional Review Board approved the study. All participants provided written informed consent in accordance with the Declaration of Helsinki.

#### Stimuli

All stimuli were pairs of words such as “hawk–carnivore,” with the first term serving as the subject term and the second term as the predicate term. Word pairs were used instead of complete sentences in order to allow for a consistent stimulus form. Using sentences would have required changes in the exact syntax of the sentence from stimulus to stimulus if awkward syntactical structures were to be avoided–compare “A carrot is a vegetable” to “Water is a beverage.” The current study used the same four lists of true stimuli used in Gruenenfelder (1986). Those lists can be found in that reference. Eighty categories were used to construct the stimuli, 20 categories per list. For each category, two true stimuli were constructed, one involving a typical exemplar, the other an atypical exemplar. Thus, each true list consisted of 40 items. Categories were randomly assigned to lists. Typical and atypical exemplars did not differ in frequency of occurrence in the language or in word length as measured by number of letters or syllables.

In false stimuli, each predicate term was used in two different stimuli in order to parallel the construction of the true stimuli. For each type of false stimulus (Anomalous, Coordinate, Cross-Category, and Reversed), two 40-item lists were made. An Anomalous false item was created by first randomly selecting two categories from a master list of 164 categories and over 1500 exemplars, subject to the restriction that the experimenter judged the two categories to be semantically unrelated^5^. One category then served as the predicate term and a randomly chosen exemplar from the other category as the subject term. A second item was then generated for the same predicate term by randomly selecting an exemplar from a third category, also judged to be unrelated to the category used in the predicate term. Coordinate stimuli were constructed by first randomly selecting a category from the master list, and then selecting three exemplars from that category. One exemplar served as the subject term in one stimulus, the second as the subject term in a second stimulus, and the third as the predicate term in both stimuli. A pair of Cross-Category false items was constructed by first selecting a category to serve as the predicate term. Two members of a category coordinate to the predicate term then served as the subject terms of the two items. Finally, Reversed false sentences were created by first randomly selecting an exemplar, with the restriction that it be an exemplar of at least two categories on the master list and that it not be used as an exemplar in a true item. That exemplar served as the predicate term. Two of its categories were then randomly selected to serve as subject terms. The same master list of categories was used when generating category terms for true items and Cross-Category and Reversed false items and hence some category terms were repeated across these different types of stimuli. The lists of false stimuli used can be found in Supplementary Appendix 1. Examples of each type of false item are shown in Table 1.

**TABLE 1:** Examples of true and false stimuli used in Experiments 1 and 2.

		Example 1	Example 2
Condition	Stimulus type	(subject–predicate)	(subject–predicate)
**True stimuli (used in experiments 1 and 2)**			
All conditions	Typical exemplar	Carrot-vegetable	
	Atypical exemplar	Onion-vegetable	
**Experiment 1: false stimuli**			
Anomalous	Anomalous false	Dacquiri-grain	Graduation-grain
Coordinate	Coordinate false	Vision-smell	Taste-smell
Cross-category	Cross-category false	Watermelon-vegetable	Olive-vegetable
Reversed	Reversed false	Aircraft-jet	Vehicle-jet
**Experiment 2: false stimuli**			
Anomalous	Category-derived anomalous	Plane-crime	Blimp-medicine
	Coordinate-derived anomalous	Television-sparrow	Washer-hawk
Coordinate	Category-derived anomalous	<See above>	
	Similar coordinates	Television-stereo	Washer-dryer
	Less similar coordinates	Television-dryer	Washer-stereo

#### Design

The two main variables of interest in Experiment 1 were type of false stimulus used (Anomalous, Coordinate, Cross-Category, or Reversed) and typicality of true exemplars (typical or atypical). Both were manipulated within-subjects. The main question of interest concerned how each type of false stimulus affected the size of the typicality effect. Hence, type of false stimulus was manipulated across separate blocks of trials. If all types of false stimuli had been intermixed in a single block, it would have been impossible to determine the effect of each type on the typicality effect. Given that there are four conditions corresponding to the four different types of false stimulus, there are 4! or 24 possible orderings of those conditions. Across the 24 participants, each ordering was used once. Each of the four lists of true items was used only once with each participant. Across participants, each list of true items was used an equal number of times with each of the four types of false stimuli. Each of the two lists of false items for a given type was used for half the participants.

#### Procedure

All conditions were presented in a single experimental session, lasting approximately 50 min. The four types of false stimuli were presented in separate blocks of trials. Instructions before each block informed the participant about the type of false stimulus to be used in that block. Each block consisted of 80 trials, 20 true items involving typical exemplars, 20 true items involving atypical exemplars, and 40 false items. The 80 trials were presented in a different random order for each participant. These 80 trials were preceded by 20 practice trials, consisting of 10 true items and 10 false items of the same type used in the remainder of the block. None of the terms used in the practice items were used in any of the test stimuli.

A trial began with the simultaneous presentation of the stimulus and predicate terms on a computer monitor. Both words appeared on the same line of the monitor, centered horizontally and vertically, with the left-hand word serving as the subject term and the right-hand word as the predicate term. Participants were instructed to determine whether the concept named by the left-hand word was a member of the category named by the right-hand word. Participants responded by pressing either the left-most (for “true” responses) or right-most (for “false” responses) button on a 6-button response box. After the response, a small white light was turned on for 500 ms above the button corresponding to the correct response. If the participant’s response was correct, the stimulus was then erased from the monitor and the next stimulus presented after a 750 ms inter-stimulus interval (ISI). If the response was incorrect, in an effort to motivate participants to perform accurately, a larger orange light in the center of the response box was turned on for 5000 ms. After that time-out, the stimulus was erased and the next stimulus presented after the 750 ms ISI. Conditions were separated by a rest period from 3 to 5 min in duration.

### Results

**FIGURE 1:**
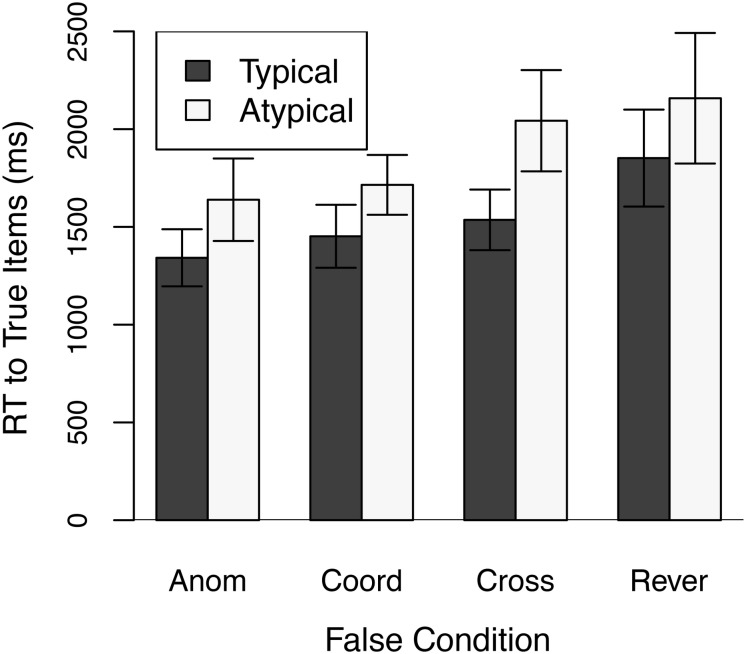
Mean Response Times (RT) in Experiment 1 to Typical and Atypical true exemplars as a function of the False condition. Anom = Anomalous False condition; Coord = Coordinate False Condition; Cross = Cross-Category False condition; and Rever = Reversed False condition. Error bars are 95% confidence intervals.

Figure 1 shows mean reaction times to correctly answered true stimuli separately for typical and atypical exemplars as a function of the type of false stimulus used. An overall repeated measures analysis of variance that included List (the four different lists of true items), Typicality, and False Condition as factors found that reaction times were slower to atypical than to typical exemplars, *F*(1,23) = 60.71, *p* < 0.001, and were affected by the type of false stimulus, *F*(3,69) = 16.50, *p* < 0.001. The Typicality x False Condition interaction was also significant, *F*(3,69) = 4.59, *p* < 0.025^6^.

**FIGURE 2:**
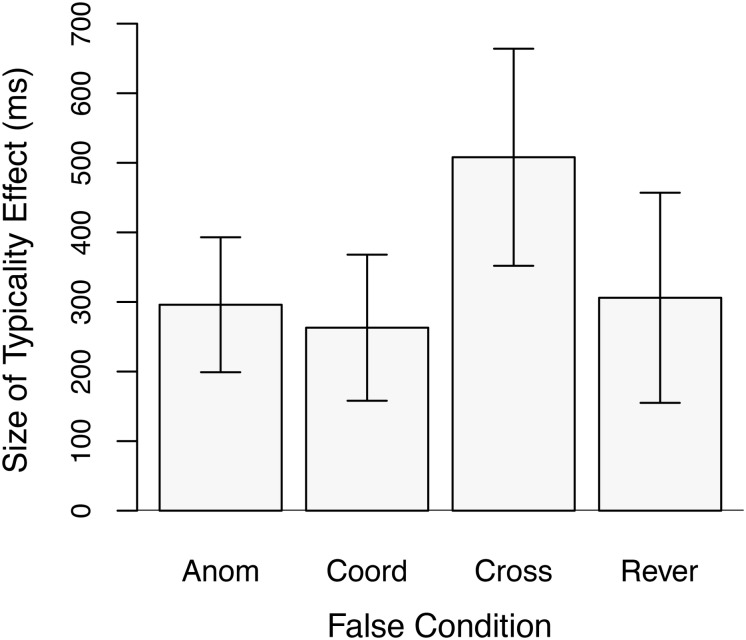
Size of the typicality effect for response times to true items in Experiment 1 as a function of False condition. Anom = Anomalous False condition; Coord = Coordinate False Condition; Cross = Cross-Category False condition; and Rever = Reversed False condition. Error bars are 95% confidence intervals.

The data of most interest concern the relative size of the typicality effect in the three conditions pairing semantically similar items in the false stimuli as compared to the size of the effect in the Anomalous condition. These data are plotted in Figure 2. A set of three planned analyses compared the size of the typicality effect in the Coordinate, Cross-Category, and Reversed conditions, respectively, to that in the Anomalous condition. Unit-information Bayes-factors (Bayes Factor or BF for short) (Rouder et al., 2009) are also reported for these analyses given the theoretical importance of null effects, i.e., the lack of an interaction between typicality and false condition. The Bayes Factor is a likelihood ratio of the relative probability of the null hypothesis (that the typicality effect is the same size in the different experimental conditions) being true given the data observed to the probability of the alternative hypothesis (of different sized typicality effects) being true. Details can be found in Rouder et al. BF_01_ is the likelihood of the null hypothesis over the alternative hypothesis, and BF_10_ is the likelihood of the alternative over the null. Results for BF_01_ are shown in Table 2. For each condition, we computed the measure twice, once with the scale factor *r* set to 1.0 and once with *r* set to 0.5. Setting *r* to a lower value, such as 0.5, is appropriate in cases where a small effect size is expected. In the present experiment, the effect size observed in the Cross-Category condition was large. If that value is used as an estimate of the effect size that could be expected in the Coordinate and Reversed conditions, then using *r* = 1.0 is quite reasonable. When evaluating the strength of the evidence favoring the null, that is the value used here. However, to allow readers to make their own judgments, results using the very conservative setting of *r* = 0.5 are also reported.

**TABLE 2:** Size of the typicality effect for true stimuli across multiple experiments and false conditions.

False Condition	Typ. effect (ms)	Typ – Typ_anom_ (ms)	*N*	*t*	Cohen’s *d*	BF_01_	
						
	(95% C. I.)	(95% C. I.)				*r* = 1.0	*r* = 0.5
**Current study: experiment 1**							
Anomalous	296 (199 – 394)	–	24	–	–	–	–
Coordinate	263 (158 – 369)	−33 (−172 – 105)	24	−0.49	−0.10	4.44	2.38
Cross-category	508 (352 – 664)	211 (122 – 301)	24	4.88	1.00	0.0016	0.0027
Reversed	306 (155 – 458)	10 (−130 – 150)	24	0.15	0.03	4.94	2.62
**McCloskey and Glucksberg (1979): experiment 2**							
Anomalous	145 (*na*)	–	16	–	–	–	–
Cross-category	307 (*na*)	162 (21 – 303)	16	2.45	*na*	0.3367	0.2801
**McCloskey and Glucksberg (1979): experiment 1**							
Anomalous	98 (*na*)	–	8	–	–	–	–
Reversed	98 (*na*)	0 (*na*)	8	0	0	3.00	1.73
**Current study: experiment 2**							
Anomalous	303 (227 – 378)	–	32	–	–	–	–
Coordinate	275 (212 – 339)	−27 (−83 – 28)	32	−1.01	−0.18	3.48	1.89
**Gruenenfelder (1986): experiment 1**							
Anomalous	277 (141 – 413)	–	18	–	–	–	–
Similar coordinate	306 (172 – 440)	29 (−109 – 167)	18	0.44	0.10	3.95	2.16
Dissimilar Coordinate	377 (260 – 494)	100 (−59 – 258)	18	1.33	0.31	1.88	1.14
**Combined analysis**							
Anomalous	291 (244 – 340)	–	92	–	–	–	–
Coordinate	298 (253 – 345)	7 (−46 – 62)	92	0.25	0.03	9.35	4.75

*Typ. effect is the typicality effect. C.I. is confidence interval. Typ – Typ_*anom*_ is the size of the typicality effect in the indicated condition minus the size of the effect in the corresponding Anomalous condition. BF_01_ is the unit-information Bayes Factor in favor of the null hypothesis over the alternative hypothesis. *na* is not available.*

**FIGURE 3:**
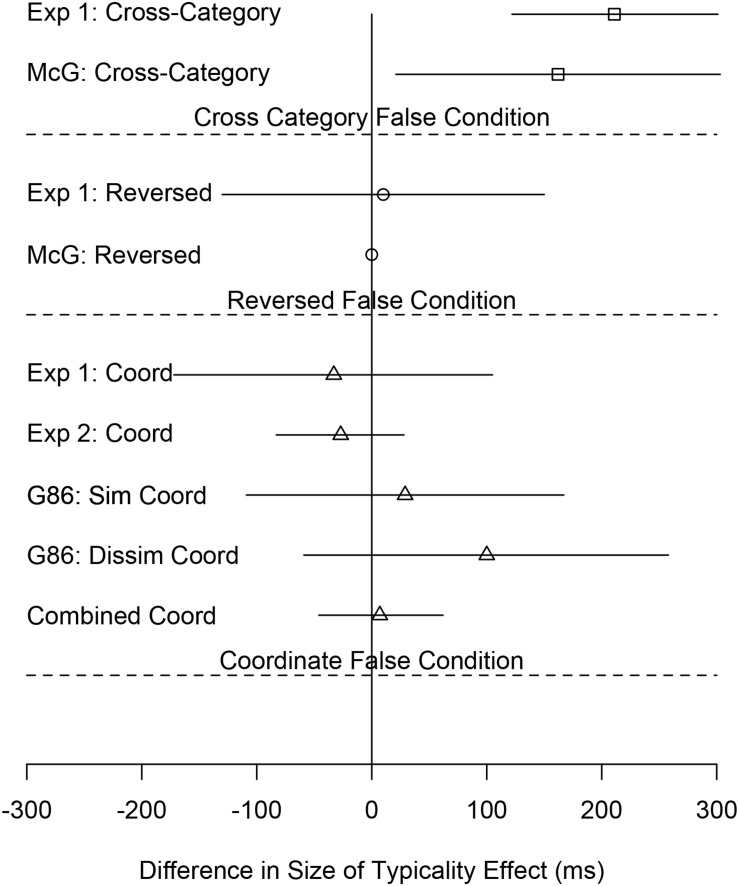
Forest plot of the size of the typicality effect in conditions pairing semantically related concepts in the false stimuli minus the size of the typicality effect in the corresponding condition pairing unrelated concepts in the false stimuli. The lines are 95% confidence intervals of the differences. Exp 1 and Exp 2 are Experiments 1 and 2, respectively, of the present study. McG are the McCloskey and Glucksberg (1979) experiments. G86 is the Gruenenfelder (1986) experiment.

Table 2 also shows the following: the size of the typicality effect and its 95% confidence interval, the change in the size of the typicality effect for each condition pairing semantically similar concepts relative to the Anomalous condition and the 95% confidence interval of that difference (Throughout this paper, the change in the size of the typicality effect refers to the size of the typicality effect in a condition pairing semantically related concepts–Coordinate, Cross-Category, or Reversed–minus the size of the typicality effect in the corresponding Anomalous condition.), the number of participants in that condition, the results of a repeated measures *t*-test comparing the size of the typicality effect to the corresponding Anomalous condition, and the effect size as measured by Cohen’s *d* for the change in the size of the typicality effect. Table 2 shows the results of multiple experiments across multiple papers. The rows corresponding to the present experiment are those under the heading “Current Study: Experiment 1.” Figure 3 shows a subset of the data in Table 2 (the change in the size of the typicality effect between false conditions using semantically related items and the corresponding Anomalous condition and the 95% confidence interval of that difference) in the form of a forest plot, also across multiple experiments. Whereas Table 2 is organized by experiment, Figure 3 is organized by false condition.

Latencies to true items were longer in the Cross-Category condition than in the Anomalous condition, *F*(1,23) = 26.73, *p* < 0.001, Cohen’s *d* = 1.05, and the increase was larger for atypical than typical exemplars, as indicated by a significant Typicality x False Condition interaction, *F*(1,23) = 23.77, *p* < 0.001, Cohen’s *d* = 1.00, BF_10_ = 625 with *r* = 1. McCloskey and Glucksberg (1979) reported sufficient data to allow a conservative approximation to the Bayes factor to be calculated for the data from their Experiment 2, which also used Cross-Category false items. Those ratios are also shown in Table 2 and are in general agreement with those from the present Experiment 1. The two studies together provide strong support for an increased typicality effect in a context of Cross-Category false items (see also Hampton, 1997).

Response times to true items were also longer in the Reversed condition than in the Anomalous condition, *F*(1,23) = 21.83, *p* < 0.001, Cohen’s *d* = 0.95. The increase, however, was approximately the same for typical and atypical true exemplars, *F*(1,23) < 1, Cohen’s *d* = 0.03. The Bayes factor indicates that the null hypothesis of equal-size typicality effects in the Reversed and Anomalous conditions is several times more likely than the alternative hypothesis of different size typicality effects. A similar result was obtained in Experiment 1 of McCloskey and Glucksberg (1979), as seen in Table 2. In both studies the effect size is at or near 0.

There was a tendency for response times to true items to be longer in the Coordinate condition than in the Anomalous condition, *F*(1,23) = 1.90, *p* = 0.18, Cohen’s *d* = 0.28. This statistically non-significant result should in no way be taken to indicate that there is no increase in response times to true items in the Coordinate condition. The increase was significant in Experiment 2 of the present study, as well as in Gruenenfelder (1986). Taking into account all three experiments, the soundest conclusion is that response times to true items do increase in the Coordinate condition. More important, similar to the case for the Reversed condition, the typicality effect was approximately the same size in the Coordinate and Anomalous conditions, *F*(1,23) < 1, Cohen’s *d* = −0.10. The Bayes factor indicates the null hypothesis of equal size typicality effects in the Coordinate and Anomalous conditions is several times more likely than the alternative hypothesis of a difference. The observed effect size is also relatively small.

Proportion correct to typical and atypical exemplars was, respectively, 0.97 and 0.87, 0.96 and 0.82, 0.95 and 0.81, and 0.89 and 0.86 in the Anomalous, Coordinate, Cross-Category, and Reversed conditions, respectively. Proportion correct to true items was slightly lower in the Coordinate condition than in the Anomalous condition, *F*(1,23) = 3.14, 0.05 < *p* < 0.10, with a slight trend toward the increase being larger for atypical than for typical exemplars, *F*(1,23) = 2.84, 0.1 < *p* < 0.2. More errors occurred to true items in the Cross-Category condition than to true items in the Anomalous condition, *F*(1,23) = 10.87, *p* < 0.005, again with a slight tendency toward a larger increase for atypical than for typical items, *F*(1,23) = 2.25, 0.1 < *p* < 0.2. More errors to true items also occurred in the Reversed condition, *F*(1,23) = 4.79, *p* < 0.05, with most of the increased errors occurring to typical items, *F*(1,23) = 7.11, *p* < 0.025. In all cases, though, the proportion correct data tracked the reaction time data–an increase in errors was accompanied by an increase in reaction time, suggesting that the reaction time results are not the consequence of a speed-accuracy trade-off.

Mean reaction times, with 95% confidence intervals in parentheses, to Anomalous, Coordinate, Cross-Category, and Reversed false items were, respectively, 1497 ms (1294 – 1700), 1466 ms (1325 – 1608), 2008 ms (1798 – 2217), and 2209 ms (1901 – 2516). Proportions correct to those four types of false stimuli were 0.98, 0.95, 0.71, and 0.86^7^, respectively. These stimuli were not constructed in a manner that allows reaction times to the different types of false stimuli to be directly compared to one another. Hence, any such comparisons can be made only with extreme caution.

### Discussion

The results of Experiment 1 provide strong support for a larger typicality effect for true items in the Cross-Category condition than in the Anomalous condition, in agreement with the results of Experiment 2 of McCloskey and Glucksberg (1979). That is, latencies to atypical exemplars increased much more in the Cross-Category condition than did latencies to typical exemplars. Experiment 1 also found moderate evidence favoring the null hypothesis of equal-size typicality effects in the Anomalous and Reversed conditions, in agreement with the results of Experiment 1 of McCloskey and Glucksberg (1979). Similarly, moderate evidence was found for equal-size typicality effects in the Anomalous and Coordinate conditions, in agreement with the results of Experiment 1 of Gruenenfelder (1986). In addition, in both the Coordinate and Reversed conditions (as well as in the Cross-Category condition), reaction times to true items were longer than in the Anomalous condition.

Reaction times to true items did not monotonically increase with the similarity of false items. In a separate experiment, participants judged the semantic similarity of the two words in each pair on a scale from 1, indicating very similar in meaning, to 6, indicating very dissimilar in meaning. Mean similarity ratings were 1.54 for typical true items, 2.71 for atypical true items, 5.92 for Anomalous items, 2.78 for Coordinate items, 3.86 for Cross-Category items, and 2.00 for Reversed items. Although Coordinate false items were judged to be more similar overall than Cross-Category false items, reaction times to true items were faster in the Coordinate condition than in the Cross-Category condition, *F*(1,23) = 12.52, *p* < 0.001, Cohen’s *d* = 0.72.

## Experiment 2

Experiment 2 had two primary purposes. The first purpose was to provide another opportunity to replicate the lack of change in the size of the typicality effect in the Coordinate condition. Reporting an attempt to replicate this null result is important because (a) it is of theoretical significance, helping to discriminate between network models and feature/distributional models, and (b) not reporting null results can result in a biased view of an effect (the file drawer phenomenon). The experiment focused on the Coordinate condition because there are both theoretical (Olson, 1970) and empirical reasons (e.g., Perfetti, 1972; Jescheniak et al., 2005) for thinking that knowledge of coordinate relations is one factor involved in both resolving reference (from a language comprehender’s perspective) and for determining which word to use to denote a particular referent (from a language producer’s perspective). Using the word “sedan,” for example, signals to the listener that the speaker is distinguishing among sedans, convertibles, sports cars and so on, whereas using the word “car” signals to the listener that the speaker is distinguishing among cars, trucks, buses and so on (see Olson, 1970). In addition, knowledge of coordinate relations makes possible some of the many seemingly effortless inferences made during language comprehension. Hearing “I walked to work today,” for example, immediately tells the listener that I did not drive, ride a bike, take the bus, or use any of the other modes of locomotion that are alternative to (i.e., coordinate to) walking.

The second purpose of Experiment 2 was to determine if the overall increase in reaction times in the Coordinate condition was due to specific semantic processing or simply reflected more general, non-specific and non-semantic processing (Kiger and Glass, 1981). Earlier work in choice reaction time (Rabbitt, 1966; Rabbitt and Rodgers, 1977; Lamming, 1979) suggests that adding more difficult items to a stimulus set can increase latencies even to easy items. Perhaps response times in the Coordinate condition are longer than in the Anomalous condition due to such overall slowing and not to any specific semantic processing.

In Experiment 2, each participant served in two conditions, each involving a category verification task. In the Anomalous condition, all false items paired unrelated concepts (Anomalous false items). In the Coordinate condition, half the false items were Coordinates and half were Anomalous false items. Across participants, the same Anomalous false items used in the Coordinate condition were also used in the Anomalous condition; these items are referred to as the critical Anomalous false items. If the increase in latencies to true items reflects semantic processes specific to discriminating category from coordinate relations, then no increase in reaction times to the critical Anomalous false items would occur in the Coordinate condition relative to the Anomalous condition. In contrast, if the increase to true items reflects an increase in general task difficulty, then latencies to the critical Anomalous false items should increase by the same amount in the Coordinate condition as do latencies to true items. Finally, the increase in latencies to true items could reflect both an increase due to semantic processes and a generalized increase due to increased task difficulty. In that case, latencies to critical Anomalous false items would increase in the Coordinate condition, but by an amount less than the increase to true items.

### Method

#### Participants

Thirty-two students from an Introductory Psychology course at Indiana University participated in the experiment in partial fulfillment of a course requirement. All reported to be native speakers of American English. The Indiana University Institutional Review Board approved the study. All participants provided written informed consent in accordance with the Declaration of Helsinki.

#### Stimuli

Two lists of true stimuli were used, each consisting of 40 typical exemplars and 40 atypical exemplars. True List 1 consisted of True Lists 1 and 2 used in Experiment 1 and True List 2 consisted of True Lists 3 and 4 from Experiment 1.

Four lists of Coordinate false items, each with 20 More Similar Coordinates (i.e., two highly related coordinate concepts) and 20 Less Similar Coordinates (i.e., two less related coordinate concepts) were also used in Experiment 2. Details of list construction, as well as the lists themselves, can be found in the Supplementary Material. Briefly, two More Similar Coordinate items were formed by pairing two typical category exemplars in one item and two atypical exemplars from the same category in a second item. Two Less Similar Coordinate items were then created from these two by interchanging the subject terms of the More Similar Coordinates. That is, if the two More Similar Coordinates for a given category were A-B (“washer–dryer”) and C-D (“television–stereo”), then the two Less Similar Coordinates were A-D (“washer–stereo”) and C-B (“television–dryer”).

From each of the two true lists, two lists of Category-Derived Anomalous false items were created by randomly re-pairing the subject and predicate terms of the true stimuli, with the restriction that no obvious relation exist between the two words of a re-paired item. Four lists of Coordinate-Derived Anomalous false items were similarly constructed from the four lists of Coordinate false items. Table 1 shows examples of the different types of false stimuli used, and indicates which types were used in the Anomalous condition and which were used in the Coordinate condition.

#### Design and Procedure

Each participant served in both the Anomalous condition and in the Coordinate condition in a single experimental session. The order of conditions was counterbalanced across participants and the two conditions were separated by a rest period approximately 3 min in duration. Each condition involved 160 trials of the category verification task presented in a random order, preceded by 20 practice trials consisting of 10 true items and 10 false items, also presented in a random order. In the practice trials, the false items were of the same type as used in the test trials for that condition. For the test trials, the true items consisted of one of the 80-item true lists. A given participant saw a different list in the two different conditions and the assignment of list to condition was counterbalanced across participants. In both conditions, 40 of the false items were Category-Derived Anomalous false items. Again, each participant saw a different list in the two conditions, and the assignment of lists to conditions was counterbalanced across participants. Furthermore, the list assigned to a given participant in a given condition was not derived from the true list used in that condition. These Category-Derived Anomalous false items, common to the two conditions across participants, served as the critical stimuli for testing the hypothesis that increased reaction times in the Coordinate condition simply reflect an increase in task difficulty as opposed to processes specific to discriminating category relations from coordinate relations. In the Coordinate condition, the remaining false items consisted of one of the lists of Coordinate false items. In the Anomalous condition, the remaining false items consisted of one of the lists of Coordinate-Derived Anomalous false items. The use of the Coordinate-Derived Anomalous false items in the Anomalous condition allowed the two conditions to have the same total number of false stimuli (40 Category-Derived Anomalous false items in each condition; plus 40 Coordinate false items in the Coordinate condition, or 40 Coordinate-Derived Anomalous false items in the Anomalous condition) and for the use of the same words in the two conditions across participants and stimuli.

As in Experiment 1, the instructions prior to each condition described the type of false stimuli to be used in that condition (though the instructions did not differentiate between Category-Derived and Coordinate-Derived Anomalous items. They were both simply described as false items pairing semantically unrelated concepts.). Half the participants made a true response with the index finger of their left hand and a false response with the index finger of their right hand. The response assignment was reversed for the other half the participants. Details of the trial-by-trial procedure, including the presentation of the stimuli, response feedback, and time-out after errors, followed those of Experiment 1.

### Results

The data of main interest are the reaction times to the critical false items, i.e., the Category Derived Anomalous false items, as a function of False Condition. The top part of Table 3 shows mean reaction times, the 95% confidence intervals around those means, and the proportion correct for correctly answered critical Category-Derived Anomalous false items separately for the Anomalous condition and the Coordinate condition. The data are broken down according to whether the items were derived from a typical or atypical category exemplar. The bottom part of the table shows the analogous data for correctly answered true items, again separately for the two conditions and for typical and atypical exemplars.

**TABLE 3:** Mean reaction times (ms) and 95% confidence intervals (95% C.I.) to the critical Category-Derived Anomalous False Stimuli and True Stimuli as a function of False Condition and typicality of the exemplar.

False condition	Typical	Atypical
**False stimuli**		
Anomalous	1272 (0.97)	1300 (0.97)
95% C.I.	1158 – 1387	1186 – 1414
Coordinate	1320 (0.98)	1326 (0.96)
95% C.I.	1208 – 1432	1213 – 1440
**True stimuli**		
Anomalous	1085 (0.97)	1388 (0.89)
95% C.I.	1003 – 1167	1235 – 1540
Coordinate	1199 (0.95)	1474 (0.85)
95% C.I.	1120 – 1278	1355 – 1593

*Numbers in parentheses are proportions correct.*

Collapsed across typicality, reaction times to the critical Category-Derived Anomalous false items were 37 ms longer in the Coordinate condition than in the Anomalous condition, a non-significant effect, *F*(1,31) < 1, Cohen’s *d* = 0.15 BF_01_ = 4.05 with *r* = 1 and 2.18 with *r* = 0.5. In contrast, reaction times to true items were longer in the Coordinate condition than in the Anomalous condition, *F*(1,31) = 8.57, *p* < 0.01, Cohen’s *d* = 0.52. Moreover, the increase in reaction times to true items in the Coordinate condition was significantly greater than the (non-significant) increase to Category-Derived Anomalous false items, *F*(1,31) = 4.93, *p* < 0.05, Cohen’s *d* = 0.39, BF_10_ = 1.71 for *r* = 1 and 2.18 for *r* = 0.5. Proportion correct to the Category-Derived Anomalous false items was approximately the same in the two conditions.

The effect of the typicality of the exemplar used in the critical Category-Derived Anomalous false items was statistically non-significant, *F*(1,31) = 1.03, Cohen’s *d* = 0.18, BF_01_ = 3.48 for *r* = 1 and 1.89 for *r* = 0.5.

As already mentioned, reaction times to true items did increase significantly in the Coordinate condition. Typical exemplars were responded to more quickly than atypical exemplars, *F*(1,31) = 84.57, *p* < 0.001. The size of the typicality effect, however, was not significantly different in the two conditions, as evidenced by the lack of a significant condition by typicality interaction, *F*(1,31) = 1.03. Odds ratios of the null to the alternative and the effect size of the difference in the size of the typicality effect are shown in Table 2 (see rows under Current Study: Experiment 2). As was the case for Experiment 1, these results offer moderate support to the hypothesis that the typicality effect is the same size in the Anomalous and Coordinate conditions. This experiment involved only two condition orders (Anomalous first versus Anomalous second). Thus it is possible to test for effects of condition order on the size of the typicality effect. There was little evidence that the typicality effect in either the Anomalous condition or in the Coordinate condition was affected by whether that condition was the first or second one administered to the participant, *t*(30) = 0.09 and *t*(30) = 0.66 for the Anomalous and Coordinate conditions, respectively.

In the Coordinate condition, mean latency to More Similar Coordinates was 1355 ms and to Less Similar Coordinates 1372 ms, a non-significant difference, *F*(1,31) < 1 (see Gruenenfelder, 2020, January 6 for additional discussion).

### Discussion

Replicating the results of earlier experiments, the typicality effect was the same size in the Coordinate condition of Experiment 2 as in the Anomalous condition.

Reaction times were not significantly longer to the critical Category-Derived Anomalous false items in the Coordinate condition than in the Anomalous condition. Although it cannot be concluded from a single experiment that there was absolutely no increase in latencies to these items in the Coordinate condition, it is the case that reaction times to true items were longer in the Coordinate condition than in the Anomalous condition, and that this increase for true items was larger than the (non-significant) increase for the critical Category-Derived Anomalous false items. This pattern of results is inconsistent with the hypothesis that the increased latencies to true items simply reflect a general increase in overall task difficulty. Rather, the increased latencies appear to reflect at least in part semantic processes necessary for discriminating category relations from coordinate relations.

The lack of an exemplar typicality effect for the critical Category-Derived Anomalous false items indicates that the reaction time differences observed for typical and atypical true stimuli cannot be accounted for by uncontrolled differences in the characteristics of the words designating typical and atypical exemplars. That is, the typicality effect observed for true stimuli appears to be due to the processing of the relation between the two words in the stimulus and not, for example, to the time to read the exemplar word.

## Combined Analysis

Experiments 1 and 2 as well as Gruenenfelder (1986) found moderate support for the null hypothesis of equal-size typicality effects for true items in the Coordinate and Anomalous conditions. An analysis that combined the data across all three of these experiments in order to increase statistical power was also conducted. Gruenenfelder (1986) included two Coordinate conditions, one using More Similar Coordinate false stimuli, the other using Less Similar Coordinate false stimuli (referred to, respectively, as Similar and Dissimilar Coordinates in the original report). Both conditions were included in the combined analysis.

The combined analysis also increases the sample size to a level where the frequency distribution of the size of the typicality effect can be meaningfully examined across the Anomalous and Coordinate conditions. Conceivably, different participants in the Coordinate conditions may have adopted different strategies, one such strategy resulting in a decreased typicality effect, the other in an increased typicality effect, such that the overall result was no change in the mean typicality effect. If so, then a bi-modal frequency distribution in the Coordinate conditions would be expected. Bi-modality *per se* can be difficult to detect statistically. However, such a strategy mix in the Coordinate condition could also show up as a simple, more easily detectable difference in the frequency distributions of the typicality effect across the Anomalous and Coordinate conditions.

### Results

The details of the combined analysis are shown in Table 2 (see the rows titled Combined Analysis) and summarized in Figure 3. The analysis considerably strengthens the evidence favoring the null hypothesis of equal-size typicality effects in the Coordinate and Anomalous conditions. The Bayes Factor favoring the null is twice as large in the combined analysis as the largest Bayes Factors from the individual experiments. In addition, the effect size, as indicated by a Cohen’s *d* of 0.03, is quite small.

**FIGURE 4:**
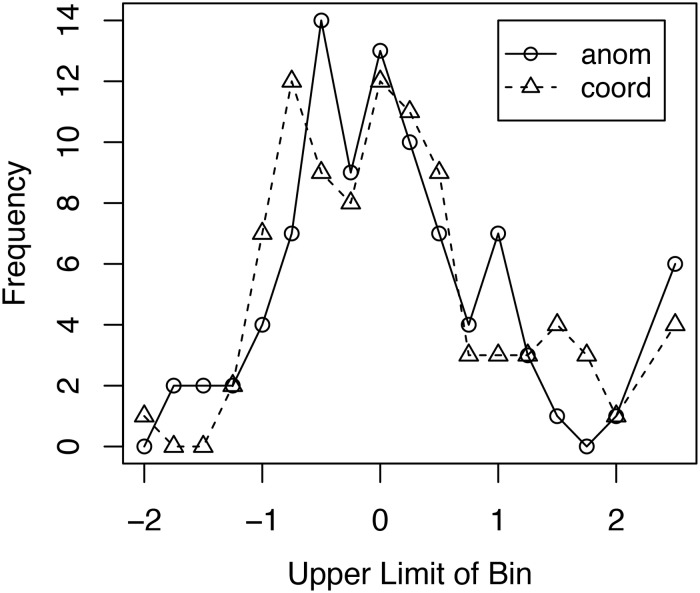
Frequency distribution of the size of the typicality effect for response times for true items for the combined analysis. Bins are in standard deviation units. The right most data points for the two curves include all response times 2.25 or more standard deviations above the mean. anom = Anomalous False conditions; coord = Coordinate False conditions.

Figure 4 shows the frequency distribution of the size of the typicality effect for the 92 participants from the three experiments/four conditions included in the combined analysis. The frequency distribution is plotted in standard deviation units. For each condition, the typicality effect was determined for each participant. The overall mean and standard deviation of the effect for that condition was then calculated across participants. The size of that participant’s typicality effect in standard deviation units was then calculated by subtracting the participant mean from that individual’s effect and dividing the result by the standard deviation for that condition. In creating the frequency distributions, a bin size of 0.25 standard deviations was used. An examination of Figure 4 shows no obvious differences in the frequency distributions for the Anomalous and Coordinate conditions. A chi-square test comparing the two distributions showed no statistically significant difference between those distributions, χ^2^(17, *N* = 92) = 15.56, *p* = 0.56. These results offer no support for the hypothesis that the invariance of the typicality effect across the Anomalous and Coordinate conditions is due to a mix of strategies in the Coordinate condition, some resulting in an increased typicality effect, others in a decreased typicality effect.

### Discussion

In the present set of studies, the Bayes Factor is being used to make judgments of the relatively likelihood of the null hypothesis (of no change in the size of the typicality effect) relative to the alternative hypothesis (of a change in the size of the effect). As can be seen in Table 2, the combined analysis provides strong support for favoring the null over the alternative when a scaling factor of 1 is used, and moderately strong support when the very conservative scaling factor of 0.5 is used. In addition, across all the studies involving Coordinate and Reversed false items reported in Table 2, with the exception of the Dissimilar Coordinate condition in Experiment 1 of Gruenenfelder (1986), the effect sizes are small. As already noted, the effect size for the combined analysis was particularly small. In the context of null hypothesis significance testing, it may also be useful to provide information concerning the statistical power of the various experiments. When calculating power, an alpha value of 0.05 or smaller is traditionally used when the hypothesis of interest predicts the alternative hypothesis. Here, the hypothesis of interest predicts a null effect and hence a value of 0.2 was used for alpha. If several variations of an experiment, in which a null effect is predicted, fail to reach a traditional level of statistical significance but do consistently find a *p-*value of less than 0.2, it would be hard to claim even moderate support for the null hypothesis.

For a small effect size of 0.2 and an alpha of 0.2, the powers of Experiments 1 and 2 and Experiment 1 of Gruenenfelder (1986) were relatively weak: 0.39, 0.44, and 0.34, respectively. None of those studies found a *p-*value of less than 0.2 when assessing changes in the size of the typicality effect (The *p-*value for the Dissimilar Coordinate condition of Gruenenfelder [1986] was just over 0.2.). For the combined analysis, the power is a more respectable 0.74. Nevertheless, there was little hint of an effect. For a moderate effect size of 0.5 (half of the actual effect size found in the Cross-Category condition of Experiment 1) and an alpha of 0.2, the powers of Experiments 1 and 2 and Experiment 1 of Gruenenfelder (1986) were 0.87, 0.93, and 0.78, respectively. The power of the combined analysis, rounded to the second decimal place, was 1. There is enough information in Table 2 for the interested reader to compute *p-*values for all these comparisons. Taken in the aggregate, the power analysis, the Bayes Factors which take into account the actually observed data as well as the sample sizes, and the actually observed effect sizes indicate that if there is a change in the size of the typicality effect in the Coordinate condition relative to the Anomalous condition, it is small.

## General Discussion

The results of the present experiments can be summarized as follows. First, in a category verification task, compared to a condition in which false stimuli pair two semantically unrelated words (Anomalous falses), reaction times to true items increase when the false items reverse the order of the category and exemplar terms (Reversed falses), when the false items pair an exemplar with a category coordinate to its proper category (Cross-Category falses), and when the false items pair two coordinates with one another (Coordinate falses). Second, the amount of that increase does not appear directly related to the semantic similarity of the two terms paired in a false item. The increase was less in the Coordinate condition than in the Cross-Category condition, even though the terms paired in Coordinate falses were more similar to one another than the terms paired in the Cross-Category falses. Third, and most important, the typicality effect for true items was larger in the Cross-Category condition than in the Anomalous condition but the same size in the Coordinate and Reversed conditions as in the Anomalous condition. It is of course extremely difficult if not impossible to definitively prove an effect size of 0. The preponderance of evidence, however, indicates that if there is a change in the size of the typicality effect in the Coordinate or Reversed conditions, it is quite small. The invariance of the typicality effect across the Anomalous, Coordinate, and Reversed conditions is consistent with the hypothesis that performance in these conditions is based on the retrieval of semantic relations from an associative network. The increased typicality effect in the Cross-Category condition is not consistent with that hypothesis.

### Semantic Network Models

The most straightforward explanation of the overall pattern of results seems to be in terms of a *sparse semantic network* model of lexical semantic memory. Where it is able to discriminate true from false stimuli in a category verification task, such as in the Coordinate and Reversed conditions, this network is used to determine the response. Where it is not able to make that discrimination, such as in the Cross-Category condition, that network is augmented by an additional representation–process pair, such as a comparison of the semantic features of the two concepts named in a stimulus.

As mentioned earlier, within a network model, verifying a stimulus in a category verification task involves attempting to retrieve an association or a chain of associations between the two terms of that stimulus. To briefly review, the retrieval process terminates when either an association or chain of associations linking the two terms is retrieved, or when all paths of associations less than or equal to a particular *stopping length* are retrieved without retrieving a chain linking the two terms. In the event of such a failure to retrieve a chain of associations linking the subject and predicate terms, the assumption is that it is relatively safe to conclude that the two terms are not semantically related (i.e., that the stimulus is an Anomalous false). Analyses of networks based on word co-occurrences suggests that the mean shortest path between arbitrarily chosen words in that network is quite short, on the order of three associations (Gruenenfelder et al., 2016; see also Kenett et al., 2017). For that reason, it is safe to set the stopping length to a small number of associations; a stopping length of 2 is assumed here. The time to perform this retrieval stage is affected by associative strength. Stronger associations are retrieved more quickly than weaker associations. Depending upon the discrimination required between true and false stimuli, this retrieval stage may be sufficient to make a response. In particular, in the Anomalous condition, where all false stimuli are Anomalous, if an associative path is retrieved between the subject and predicate terms, a true response is made. If no such path can be retrieved within the stopping length, a false response is made.

In the Coordinate and Reversed conditions, retrieving an associative path linking the two terms in the stimulus is necessary but does not by itself discriminate true from false stimuli. Retrieval of such an associative path is possible for both true and false stimuli. Hence, the retrieval process must be followed by the path evaluation process that uses the labels of the retrieved associations. Given a stopping length of 2 associations, the path evaluation process is assumed to use 2-tuples, where the first element of the tuple indicates the label on the first association in the retrieved chain and the second element indicates the label on the second association in the retrieved chain. In the event that a direct association is retrieved between the subject and predicate terms of a stimulus, the second element is the null relation, symbolized here as NULL. In the Coordinate and Reversed conditions, for the retrieval process beginning with the subject term, the tuples (COORD, NULL) and (COORD, COORD), for instance, would in these conditions indicate that the stimulus is a coordinate relation. The tuples (SUBSET, NULL) or (COORD, SUBSET) would indicate a subset–superset relation, See the Supplementary Material for an exhaustive mapping of such 2-tuples to type of semantic relation. Each possible 2-tuple forms a production rule that maps to a decision value of either true or false. Hence, the path evaluation process is akin to indexing into a table with the 2-tuple and determining the associated decision value. There is no motivated reason for assuming execution of any one production rule is favored over any other, and hence to assume that the path evaluation process is affected by typicality.

To be sure, some retrieved associative paths can be ambiguous with respect to category verification. For example, consider the true stimulus robin–bird in the Reversed condition. One possible retrieved path is robin–animal–bird (SUBSET, SUPERSET). For the false stimulus, bird–robin, one possible path is bird–animal–robin (also SUBSET, SUPERSET). That is, a retrieved associative path consisting of a subset relation followed by a superset relation in the Reversed and Coordinate conditions is ambiguous concerning the truth of the sentence. Several ways of participants’ dealing with ambiguous paths are discussed in the Supplementary Material. The gist of that discussion is that it is very unlikely that such paths are retrieved frequently enough to affect overall mean response times, and hence the size of the typicality effect.

In the case of the Cross-Category condition, the sparseness of the network makes it unreliable for discriminating true from false responses–all retrieved associative chains are ambiguous. Because an exemplar may form an association with an inappropriate category, and because coordinate relations may cross category boundaries, the labels on the chain of associations linking the two terms of a false stimulus may be the same as those on the chain linking the two terms of a true stimulus. Consider the false stimulus *pretzel–beverage*. *Cola* may first be retrieved from *pretzel*, and *beverage* then retrieved from *cola*, perhaps leading to the false conclusion that the stimulus is true. Consequently, in the Cross-Category condition, participants must use some other strategy, such as determining the similarity of the subject and predicate terms, in order to determine the truth of the stimulus.

This sparse network model, including suggested paths toward its falsification as well as a brief discussion of how it may develop, is discussed in more detail in the Supplementary Material.

### Feature and Distributional Models

The fact that overall response times to true items were longer in the Cross-Category condition than in the Coordinate condition, despite the fact that the overall similarity of Cross-Category false items was less than that of Coordinate items, argues against feature models in which performance in the category verification task reflects a simple feature comparison process (or a comparison of vector values in a distributional model) that calculates similarity. The invariance of the typicality effect across the Anomalous, Coordinate, and Reversed conditions also argues against such models. That result would also seem hard to predict, without positing a coincidence, by more complex models in which different semantic relations are discriminated from one another by examining different sets of features, as described in the Introduction. This result should not, however, be interpreted as indicating that feature models or distributional models have no role to play in lexical semantic memory. They undoubtedly do. In fact, just in terms of the present experiments, it would be difficult to obtain above-chance performance in the Cross-Category condition without relying on some such model. It is also quite possible that as the ability of these models to represent different semantic relations is explored in more detail, a representation–process pair can be found that predicts equal-sized typicality effects across the Anomalous, Coordinate, and Reversed conditions, or that predicts a very small change in the size of that effect. Such a development would be welcome, as it would undoubtedly advance our understanding of lexical semantic memory.

There is in fact good reason to be optimistic in this respect, especially with regards to distributional models. In terms of constructing their vectors, distributional models begin with the same raw data as do network models–the pattern of co-occurrences of pairs of words. That co-occurrence is then transformed into vector representations unique to the particular model. Those vectors typically retain some information about which words co-occur together (i.e., would be linked by an association in a network model), in that words that co-occur are going to have some similar vector values. If somehow the vector also retains information about the syntactical context in which the words co-occur, then the raw ingredients are there to determine not simply that two words are related, but also to determine more precisely the semantic relation between them.

One approach to category verification using feature representations that has not yet been discussed in the present paper is what might be dubbed the *feature inclusion hypothesis*. If X possesses all the features of Y plus some additional ones, then X is an exemplar of the category Y. This approach is quite similar to the defining feature approach of Smith and colleagues (Rips et al., 1973; Smith et al., 1974b). In some circumstances, such as in the Anomalous and Cross-Category conditions (at least for typical true exemplars), a fast similarity computation may be sufficient for discriminating true from false stimuli. In other situations, such as the Coordinate and Reversed conditions, where similarity does not discriminate true from false stimuli, only these inclusive features are examined. Although rejecting such an approach may be premature, it does have several difficulties. First, many exemplars bear more of a family resemblance to their category than a set of defining features. Second and related, in a sense this approach requires perfect knowledge of features. A person may know that squirrels are mammals without ever having explicitly learned squirrels bear their young live. Third, it is unclear whether this approach offers a motivated reason for equal-size typicality effects across the Anomalous, Coordinate, and Reversed conditions.

### Alternatives and Limitations

One possible alternative to the network model is that in the Coordinate and Reversed conditions participants base their responses on the hierarchical level of the two terms in the stimulus. In the Coordinate condition, if the two terms are at the same hierarchical level, respond “false;” if they are at different hierarchical levels, respond “true.” In the Reversed condition, determine whether the first or second word is at the higher level and respond accordingly. Empirically, this hypothesis has difficulty explaining why a typicality effect occurs at all in these two conditions. Is hierarchical level somehow more difficult to determine for atypical exemplars? More theoretically, hierarchical level seems to be more a property of the relation between two words than of a word in and of itself. Intuitively, it is easy to see that penguin and sparrow are at the same level of generality and that bird is at a higher level. When words are not taxonomically related, however, such judgments seem more difficult. Is *German Shepherd* at the same level of generality as *sparrow* or as *chipping sparrow*? Is it at the same level of generality as *sedan* or as *Chevrolet Impala*? It would seem that information about level is not a property of the word itself but is derived from the relation between two words. If that relation is set-superset, the two words are at different levels. If it is a coordinate relation, then they are at the same level. In other words, arguing that participants in the Coordinate and Reversed conditions are making their judgments based on the relative hierarchical level of the two terms in the item seems to be the same as saying that they determine their responses by first determining if the two words are in a set-superset or coordinate relation. Determining that relation, though, is sufficient to then respond without continuing to determine the hierarchical level of the two words.

A fair question to ask of the network model, is whether coordinate relations are required. Could all the necessary taxonomic relations be represented using only SUBSET and SUPERSET relations? Could one not determine that two items are coordinate to one another if they share the identical set of subset relations? It is reasonable to infer that if two items do share an identical set of subset relations, then they are coordinates. However, there would seem to be several problems with this approach. First, it would seem to require a very dense network of subset relations. If *parrot* has no subset relation to *bird*, but *dove* does, how could we know that *parrot* and *dove* are coordinates? The pattern of co-occurrences within language seems to argue against such a dense network–exemplars do not necessarily occur frequently enough with a particular category term to form an association (Shwartz et al., 2016). Second, if as suggested earlier, coordinate information is used to make some of those apparently effortless inferences routinely made in the course of language comprehension, then an efficient means of determining whether two concepts are coordinate would seem to be desirable. Being able to retrieve directly from a word its important set of coordinates would seem to meet this condition better than having to retrieve all the subset links from a term, and then all the subset links from all those terms to determine which in fact are coordinates (Note that the fact that two concepts simply share a subset relation with a third concept does not make them coordinates. *Dog* and *mammal* both share a subset relation with *animal*, but *dog* and *mammal* are not coordinate). Third, not all concepts that reasonably seem to be in a coordinate relation share all their subset links. *Chicken* and *pork*, for instance, would seem to be coordinate concepts, and both have subset links to *meat*. *Chicken*, however, also has subset links to *poultry* and *bird* which *pork* does not share. Fourth, in a network model with no coordinate associations, and hence no coordinate relations that are crossing category boundaries, it is not clear why performance in the Cross-Category condition should cause any difficulty at all. In brief, this approach to representing coordinate information has a number of obstacles to overcome.

The network model described here does not currently contain what might be called NOT_A relations. For some exemplars, people may specifically learn, by being specifically told, that some exemplars are not members of a particular category. In American English, “Tomatoes are not vegetables,” “Bats are not birds,” and “Whales are not fish” come to mind. Hearing such statements could conceivably lead to specifically encoding those relations, e.g., “NOT_A(bat, bird). Retrieving such a relation could lead to determining that certain stimuli are false in the category verification task. Retrieving NOT_A(bat, bird) is sufficient in, for example, the Cross-Category condition, to reject as false the stimulus “bat–bird.” The existence of such relations is a real possibility. However, there may very well be few such relations. In American English, people may explicitly hear that tomatoes are not vegetables but few if any have probably explicitly heard that peaches are not vegetables. Presumably, if such NOT_A relations were prevalent, performance may well have been much better in the Cross-Category condition of Experiment 1. Nevertheless, the role of such NOT_A relations in semantic memory may well be a fruitful area of future work.

## Conclusion

Theories of lexical semantic memory require some mechanism for determining the semantic relation between pairs of words. The nature of the mechanism is one characteristic that distinguishes various classes of these theories. In network models, semantic relations are directly encoded via the labels on the associations; in feature and distributional theories, those relations are derived or computed by comparing the features of the two terms or their values on the dimensions making up the space (Smith, 1978). The present paper provides support for the direct encoding of at least category and coordinate relations in a semantic network. That network is sparse–not all exemplar-category pairs are linked by a category relation and not all coordinate terms are linked by a coordinate relation. The present model handles the issue of sparseness by relying on the transitivity of certain semantic relations–if A is a subset of B and B of C, then A is also a subset of C. If A is a coordinate of B and B of C, then A is also a coordinate of C. Baroni et al. (2010) have shown that it is possible to derive many semantic relations by analyzing the syntactical patterns in which words co-occur (see also Nguyen et al., 2017b). The extent to which those additional relations are also represented in the mind as semantic networks is a topic for future research.

The network model presented here is not meant as a complete model of lexical semantic memory. It simply does not seem able to account for the full richness of humans’ knowledge and use of word meanings. The network is merely one component of a complete model, and that complete model very likely includes additional representations, such as feature representations or distributional/spatial representations (for other hybrid accounts of semantic memory, see, for example, Lorch, 1981; Hampton, 1997; McRae et al., 1997; Louwerse, 2011; Murphy et al., 2012). With respect to the present study alone, a second component appears necessary to explain the results from the Cross-Category condition. Such hybrid, multi-component models can be difficult to falsify unless the role of the different components is clearly delineated. The suggestion here is that the semantic network component is used for representing the specific semantic relation that exists between various pairs of words.

## Notes

(1) This approach has similarities to the two-stage feature model of Smith et al. (1974a, b), both in spirit and in specific possible implementations.

(2) The studies cited in this paragraph were attempts to extract specific semantic relations, such as set–superset, superset–set, and coordinate relations–from distributional representations. They were not intended as models of human performance, and the comments here should thus not be misconstrued as criticisms of this work. Rather the comments simply indicate that the obvious extension of these approaches to human performance may encounter some empirical challenges. Further, these models are not necessarily incompatible with the network model described below. The distributional model may serve as an interim representation that is used, perhaps in conjunction with syntactical pattern analyses (Nguyen et al., 2017b) to derive the semantic relation existing between pairs of words. That semantic relation could then be encoded within an associative network. Such an approach is one way to deal with the issue of sparseness, i.e., the fact that most pairs of words do not co-occur frequently enough to derive the semantic relation between them, relying entirely on associative learning and syntactical pattern analysis (Shwartz et al., 2016). That approach contrasts with the one taken by the network model described later, in which the sparseness problem is dealt with by taking advantage of the (near) transitivity of some semantic relations.

(3) It is perhaps worth mentioning that some theorists have included an associative network as part of lexical memory, but with unlabeled associations (Lucas, 2000; Hutchison, 2003; Wettler et al., 2005; Maki and Buchanan, 2008). These associations link the phonological and/or orthographic form of words but carry no information regarding the nature of the semantic relation between those words. For additional discussion, see McRae et al. (2012).

(4) Another way of viewing the invariance of the typicality effect in the Coordinate and Reversed conditions, in comparison with the Anomalous condition, is through the lens of Sternberg’s additive factors methodology (Sternberg, 1969). According to this approach, if two factors have additive effects on reaction time, then they likely affect different stages of processing. If they interact, they likely affect the same stage of processing. Here, typicality and type of false stimuli (when considering the Coordinate and Reversed conditions) have additive effects, suggesting that they affect different stages of processing. Hence, if typicality affects retrieval, that leaves path evaluation to be affected by type of false stimulus. And if path evaluation is affected by type of false stimulus, the additivity of typicality and type of false stimulus, indicates that typicality does not affect path evaluation.

(5) An initial master list of categories and exemplars (with typicality ratings) was first constructed using previously collected category norms. Because of the large number of category terms needed for this experiment, that list was then augmented with additional categories and exemplars generated by the author. Typicality ratings for the exemplars of those additional categories were also collected (see Gruenenfelder, 1984).

(6) Neither the main effect of List nor any of its interactions with the other factors approached significance. In such a case, and given that, across subjects, the same true items were used in all four False Conditions, an appropriate way to analyze the data is to treat only subjects as a random effect, rather than conducting quasi-F or min-F’ tests (Raaijmakers et al., 1999; Raaijmakers, 2003). Further, a null effect, in particular the lack of an interaction between Typicality and False Condition, was of major theoretical interest in the present study. Hence tests that are more likely to commit a Type I error are favored over more conservative tests. This factor too argues for analyses using only subjects as a random factor. Accordingly, that was the approach followed in the present study. A similar comment applies to both the true and false stimuli of Experiment 2. Analyses were also done in which latencies greater than 2.5 standard deviations above a participant’s mean latency in a given condition were dropped. This procedure affected fewer than 1.5% of the responses and resulted in no qualitative differences in the results. The datasets used in the analyses have been deposited with the Open Science Foundation (OSF), https://dx.doi.org/10.17605/OSF.IO/ZGMH7.

(7) The low percentage correct to Cross-Category false items suggests that with a higher percentage correct, the increase in the typicality effect observed in this condition would have been even larger. In addition, this result in no way mitigates the need for an explanation of the finding of no change in the size of the typicality effect in the Coordinate and Reversed conditions.

## Data Availability Statement

The data underlying the analyses of both experiments as well as the combined analysis have been deposited with the Open Science Framework and are available at https://dx.doi.org/10.17605/OSF.IO/ZGMH7.

## Ethics Statement

The studies involving human participants were reviewed and approved by Institutional Review Board, Indiana University Bloomington. The patients/participants provided their written informed consent to participate in this study.

## Author Contributions

TG: substantial contributions to conception and design, acquisition of data, analysis and interpretation of data, drafting the article and revising it critically for important intellectual content, and final approval of the version to be published.

## Conflict of Interest

The author declares that the research was conducted in the absence of any commercial or financial relationships that could be construed as a potential conflict of interest.

## References

[B1] Baroni M. Bernardi R. Do N.-Q. Shan C.-C. (2012). “Entailment above the word level in distributional semantics,” in *Proceedings of the 13th Conference of the European Chapter of the Association for Computational Linguistics*, Avignon, 23–32.

[B2] Baroni M. Murphy B. Barbu E. Poesio M. (2010). Strudel: a corpus-based semantic model based on properties and types. *Cogn. Sci.* 34 222–254. 10.1111/j.1551-6709.2009.01068.x 21564211

[B3] Burgess C. (1998). From simple associations to the building blocks of language: modeling meaning in memory with the HAL model. *Behav. Res. Methods Instrum. Comput.* 30 188–198. 10.3758/bf03200643

[B4] Chaffin R. J. S. Herrmann D. J. (1981). Comprehension of semantic relationships and the generality of categorization models. *Bull. Psychon. Soc.* 17 69–72. 10.3758/bf03333670

[B5] Chaffin R. J. S. Herrmann D. J. (1984). The similarity and diversity of semantic relations. *Mem. Cogn.* 12 134–141. 10.3758/bf03198427 6727635

[B6] Church K. W. Hanks P. (1990). Word association norms, mutual information, and lexicography. *Comput. Linguist.* 16 22–29.

[B7] Collins A. M. Quillan M. R. (1969). Retrieval time from semantic memory. *J. Verbal Learning Verbal Behav.* 8 240–247. 10.1016/s0022-5371(69)80069-1

[B8] Cree G. S. McNorgan C. McRae K. (2006). Distinctive features hold a privileged status in the computation of word meaning: implications for theories of semantic memory. *J. Exp. Psychol. Learn. Mem. Cogn.* 32 643–658. 10.1037/0278-7393.32.4.643 16822138 PMC3226832

[B9] Gellatly A. R. H. Gregg V. H. (1975). The effects of negative relatedness upon word-picture and word-word comparisons and subsequent recall. *Br. J. Psychol.* 66 311–323. 10.1111/j.2044-8295.1975.tb01467.x 1182400

[B10] Gellatly A. R. H. Gregg V. H. (1977). Intercategory distance and categorization times: effects of negative-probe relatedness. *J. Verbal Learning Verbal Behav.* 16 505–518. 10.1016/s0022-5371(77)80043-1

[B11] Glass A. L. Holyoak K. J. (1975). Alternative conceptions of semantic theory. *Cognition* 3 313–339. 10.1016/0010-0277(74)90002-x

[B12] Gruenenfelder T. M. (1984). Typicality ratings for 893 exemplars of 93 categories. *Behav. Res. Methods Instrum. Comput.* 16 351–354. 10.3758/bf03202461

[B13] Gruenenfelder T. M. (1986). Relational similarity and context effects in category verification. *J. Exp. Psychol. Learn. Mem. Cogn.* 12 587–599. 10.1037/0278-7393.12.4.587

[B14] Gruenenfelder T. M. (2020). *Interference Can Affect Response Times to False Items in Category Verification.* Bloomington, IN: Indiana University.

[B15] Gruenenfelder T. M. Recchia G. Rubin T. Jones M. N. (2016). Graph-theoretic properties of networks based on word association norms: implications for models of lexical semantic memory. *Cogn. Sci.* 40 1460–1495. 10.1111/cogs.12299 26453571

[B16] Hampton J. A. (1997). Associative and similarity-based process in categorization decisions. *Mem. Cogn.* 25 625–640. 10.3758/bf03211304 9337581

[B17] Hino Y. Pexman P. M. Lupker S. J. (2006). Ambiguity and relatedness effects in semantic tasks: are they due to semantic coding? *J. Mem. Lang.* 55 246–273. 10.1016/j.jml.2006.04.001

[B18] Holyoak K. J. Glass A. L. (1975). The role of contradictions and counterexamples in the rejection of false sentences. *J. Verbal Learning Verbal Behav.* 14 215–239. 10.1016/s0022-5371(75)80066-1

[B19] Hutchison K. A. (2003). Is semantic priming due to association strength or feature overlap? A *micro*analytic review. *Psychon. Bull. Rev.* 10 785–813. 10.3758/s13423-018-1453-6 15000531

[B20] Jaccard P. (1912). The distribution of the flora in the Alpine Zone. *New Phytol.* 11 37–50. 10.1111/j.1469-8137.1912.tb05611.x

[B21] Jescheniak J. D. Hantsch A. Schriefers H. (2005). Context effects on lexical choice and lexical activation. *J. Exp. Psychol. Learn. Mem. Cogn.* 31 905–920. 10.1037/0278-7393.31.5.905 16248741

[B22] Jones M. N. Mewhort D. J. K. (2007). Representing word meaning and order information in a composite holographic lexicon. *Psychol. Rev.* 114 1–37. 10.1037/0033-295X.114.1.1 17227180

[B23] Kenett Y. N. Levi E. Anaki D. Faust M. (2017). The semantic distance task: quantifying semantic distance with semantic network path length. *J. Exp. Psychol. Learn. Mem. Cogn.* 43 1470–1489. 10.1037/xlm0000391 28240936

[B24] Kiger J. I. Glass A. L. (1981). Context effects in sentence verification. *J. Exp. Psychol. Hum. Percept. Perform.* 7 688–700. 10.1037/0096-1523.7.3.688

[B25] King D. R. Anderson J. R. (1976). Long-term memory search: an intersecting activation process. *J. Verbal Learning Verbal Behav.* 15 587–605. 10.1016/0022-5371(76)90053-0

[B26] Lamming D. R. J. (1979). Choice reaction performance following an error. *Acta Psychol.* 43 199–224. 10.1016/0001-6918(79)90026-x

[B27] Landauer T. K. Dumais S. T. (1997). A solution to Plato’s problem: the latent semantic analysis theory of acquisition, induction, and representation of knowledge. *Psychol. Rev.* 104 211–240. 10.1037/0033-295x.104.2.211

[B28] Lenci A. Benotto G. (2012). “Identifying hypernyms in distributional semantic spaces,” in *Proceedings of the First Joint Conference on Lexical and Computational Semantics*, Montréal, 75–79.

[B29] Link S. W. (1975). The relative judgment theory of two choice reaction time. *J. Math. Psychol.* 12 114–136.

[B30] Lorch R. F. J. (1981). Effects of relation strength and semantic overlap in retrieval and comparison processes during sentence verification. *J. Verbal Learning Verbal Behav.* 20 593–610. 10.1016/s0022-5371(81)90193-6

[B31] Lorch R. F. J. (1982). Priming and search processes in semantic memory: a test of three models of semantic activation. *J. Verbal Learning Verbal Behav.* 21 468–492. 10.1016/s0022-5371(82)90736-8

[B32] Louwerse M. (2011). Symbol interdependency in symbolic and embodied cognition. *Top. Cogn. Sci.* 3 273–302. 10.1111/j.1756-8765.2010.01106.x 25164297

[B33] Lucas M. (2000). Semantic priming without association: a meta-analytic review. *Psychon. Bull. Rev.* 7 618–630. 10.3758/bf03212999 11206202

[B34] Lund K. Burgess C. (1996). Producing high-dimensional semantic spaces from lexical co-occurrence. *Behav. Res. Methods Instrum. Comput.* 28 203–208. 10.3758/bf03204766

[B35] Maki W. S. Buchanan E. (2008). Latent structure in measures of associative, semantic, and thematic knowledge. *Psychon. Bull. Rev.* 15 598–603. 10.3758/pbr.15.3.598 18567261

[B36] Masson M. E. J. (1995). A distributed memory model of semantic priming. *J. Exp. Psychol. Learn. Mem. Cogn.* 21 3–23. 10.1037/0278-7393.21.1.3

[B37] McCloskey M. Glucksberg S. (1979). Decision processes in verifying category membership statements: implications for models of semantic memory. *Cogn. Psychol.* 11 1–37. 10.1016/0010-0285(79)90002-1

[B38] McRae K. Cree G. S. Seidenberg M. S. McNorgan C. (2005). Semantic feature production norms for a large set of living and non-living things. *Behav. Res. Methods Instrum. Comput.* 37 547–559. 10.3758/bf03192726 16629288

[B39] McRae K. de Sa V. R. Seidenberg M. S. (1997). On the nature and scope of featural representation of word meaning. *J. Exp. Psychol. Gen.* 126 99–130. 10.1037/0096-3445.126.2.99 9163932

[B40] McRae K. Khalkhali S. Hare M. (2012). “Semantic and associative relations: examining a tenuous dichotomy,” in *The Adolescent Brain: Learning, Reasoning, and Decision Making*, eds Reyna V. F. Chapman S. Dougherty M. Confrey J. (Washington, DC: American Psychological Association), 39–66. 10.1037/13493-002

[B41] Mikolov T. Sutskever I. Chen K. Corrado G. Dean J. (2013). Distributed representations of words and phrases and their compositionality. *Adv. Neural Inf. Process. Syst.* 26 3111–3119. 10.1098/rstb.2019.0305 31840584 PMC6939345

[B42] Moss H. E. Hare M. L. Day P. Tyler L. K. (1994). A distributed memory model of the associative boost in semantic priming. *Conn. Sci.* 6 413–427. 10.1080/09540099408915732

[B43] Murphy G. L. Hampton J. A. Milovanovic G. S. (2012). Semantic memory redux: an experimental test of hierarchical category representation. *J. Mem. Lang.* 67 521–539. 10.1016/j.jml.2012.07.005 23243336 PMC3519438

[B44] Nguyen K. A. Köper M. Schulte im Walde S. Vu N. T. (2017a). “Hierarchical embeddings for hypernymy detection and directionality,” in *Proceedings of the 2017 Conference on Empirical Methods in Natural Language Processing*, Copenhagen.

[B45] Nguyen K. A. Schulte im Walde S. Vu N. T. (2017b). “Distinguishing antonyms and synonyms in a pattern-based neural network,” in *Proceedings of the 15th Conference of the European Chapter of the Association for Computational Linguistics*, Valencia.

[B46] Nguyen K. A. Schulte im Walde S. Vu N. T. (2016). “Integrating distributional lexical contrast into word embeddings for antonym-synonym distinction,” in *Proceedings of the 54th Annual Meeting of the Association for Computational Linguistics*, Berlin.

[B47] Olson D. R. (1970). Language and thought: aspects of a cognitive theory of semantics. *Psychol. Rev.* 77 257–273. 10.1037/h0029436 5448408

[B48] Perfetti C. A. (1972). Psychosemantics: some cognitive aspects of structural meaning. *Psychol. Bull.* 78 241–259. 10.1037/h0033172

[B49] Pexman P. M. Holyk G. G. Monfils M.-H. (2003). Number-of-features effects and semantic processing. *Mem. Cogn.* 31 842–855. 10.1016/j.cognition.2014.01.001 14651293

[B50] Pexman P. M. Lupker S. J. Hino Y. (2002). The impact of feedback semantics in visual word recognition: number of features effects in lexical decision and naming tasks. *Psychon. Bull. Rev.* 9 542–549. 10.3758/bf03196311 12412895

[B51] Raaijmakers J. G. W. (2003). A further look at the “language-as-fixed-effect fallacy”. *Can. J. Exp. Psychol.* 57 141–151. 10.1037/h0087421 14596473

[B52] Raaijmakers J. G. W. Schrijnemakers J. M. C. Gremmen F. (1999). How to deal with the “the language-as-fixed-effect fallacy”: common misconceptions and alternative solutions. *J. Mem. Lang.* 41 416–426. 10.1006/jmla.1999.2650

[B53] Rabbitt P. M. A. (1966). Errors and error-corrections in choice-response tasks. *J. Exp. Psychol.* 71 264–272. 10.1037/h0022853 5948188

[B54] Rabbitt P. M. A. Rodgers B. (1977). What does a man do after he makes an error? An analysis of response programming. *Q. J. Exp. Psychol.* 29 727–743. 10.7860/JCDR/2014/7740.4157 24783127 PMC4003632

[B55] Ratcliff R. (1978). A theory of memory retrieval. *Psychol. Rev.* 85 59–108. 10.1037/0033-295x.85.2.59

[B56] Ratcliff R. Rouder J. N. (1998). Modeling response times for two-choice decisions. *Psychol. Sci.* 9 347–356. 10.1111/1467-9280.00067

[B57] Rips L. J. Shoben E. J. Smith E. E. (1973). Semantic distance and the verification of semantic relations. *J. Verbal Learning Verbal Behav.* 12 1–20. 10.1016/s0022-5371(73)80056-8

[B58] Roller S. Erk K. Boleda G. (2014). “Inclusive yet selective: supervised distributional hypernymy detection,” in *Proceedings of COLING 2014, the 25th International Conference on Computational Linguistics: Technical Papers*, Dublin, 1025–1036.

[B59] Rosch E. H. (1975). Cognitive representations of semantic categories. *J. Exp. Psychol. Gen.* 104 192–233. 10.1037/0096-3445.104.3.192

[B60] Rouder J. N. Speckman P. L. Sun D. Morey R. D. Iverson G. (2009). Bayesian t tests for accepting and rejecting the null hypothesis. *Psychon. Bull. Rev.* 16 225–237. 10.3758/PBR.16.2.225 19293088

[B61] Shwartz V. Goldberg Y. Dagan I. (2016). “Improving hypernymy detection with an integrated path-based and distributional method,” in *Proceedings of the 54th Annual Meeting of the Association for Computational Linguistics*, Berlin.

[B62] Smith E. E. (1978). “Theories of semantic memory,” in *Handbook of Learning and Cognitive Processes*, Vol. 6 ed. Estes W. K. (Hillsdale, NJ: Erlbaum).

[B63] Smith E. E. Rips L. J. Shoben E. J. (1974a). “Semantic memory and psychological semantics,” in *The Psychology of Learning and Motivation*, Vol. 8 ed. Bower G. H. (New York, NY: Academic Press).

[B64] Smith E. E. Shoben E. J. Rips L. J. (1974b). Structure and process in semantic memory: a featural model for semantic decisions. *Psychol. Rev.* 81 214–241. 10.1037/h0036351

[B65] Sternberg S. (1969). The discovery of processing stages: extensions of Donders’ method. *Acta Psychol.* 30 276–315. 10.1016/0001-6918(69)90055-9

[B66] Weeds J. Clarke D. Reffin J. Weir D. Keller B. (2014). “Learning to distinguish hypernyms and co-hyponyms,” in *Proceedings of COLING 2014, the 25th International Conference on Computational Linguistics: Technical Papers*, Dublin, 2249–2259.

[B67] Wettler M. Rapp R. Sedlmeier P. (2005). Free word associations correspond to contiguities between words in text. *J. Quant. Linguist.* 12 111–122. 10.1080/09296170500172403

