# Supplementary material for: The Representation of Coordinate Relations in Lexical Semantic Memory
Source: Front Psychol. 2020 Feb 11;11:98. doi: 10.3389/fpsyg.2020.00098 (PMC7026369; doi:10.3389/fpsyg.2020.00098)
Supplement: Supplementary file 1 [file Data_Sheet_1.PDF]

The Representation of Coordinate Relations in Lexical Semantic Memory

Thomas M. Gruenenfelder[1]

[1] Department of Psychological and Brain Sciences, Indiana University

***Correspondence:**
Thomas M. Gruenenfelder
tgruenen@indiana.edu

This Supplementary Material consists of six parts. The first two detail how the false stimuli in Experiments 1 and 2, respectively, were constructed. The third provides more detailed discussion on the sparse network model than is provided in the main paper. The fourth part briefly states the reason why, for purposes of making predictions in the experiments described in the main paper, distributional models were treated as feature models. The fifth part, Appendix 1, lists the false stimuli used in Experiment 1. The sixth part, Appendix 2, lists the coordinate false stimuli used in Experiment 2. The true stimuli used in both experiments can be found in Gruenenfelder (1986).

Experiment 1: Construction of False Stimuli

In false stimuli, each predicate term was used in two different stimuli in order to parallel the construction of the true stimuli. For each type of false stimulus (Anomalous, Coordinate, Cross-Category, and Reversed), two 40-item lists were made. An Anomalous false item was created by first randomly selecting two categories from a master list of 164 categories, subject to the restriction that the experimenter judged the two categories to be semantically unrelated. One category then served as the predicate term and a randomly chosen exemplar from the other category as the subject term. A second item was then generated for the same predicate term by randomly selecting an exemplar from a third category, also judged to be unrelated to the category used in the predicate term. Coordinate stimuli were constructed by first randomly selecting a category from the master list, and then selecting three exemplars from that category. One exemplar served as the subject term in one stimulus, the second as the subject term in a second stimulus, and the third as the predicate term in both stimuli. A pair of Cross-Category false items was constructed by first selecting a category to serve as the predicate term. Two members

of a category coordinate to the predicate term then served as the subject terms of the two items. Finally, Reversed false sentences were created by first randomly selecting an exemplar, with the restriction that it be an exemplar of at least two categories on the master list. That exemplar served as the predicate term. Two of its categories were then randomly selected to serve as subject terms. The lists of false stimuli used can be found in Appendix 1.

Experiment 2: Construction of False Stimuli

Four lists of Coordinate false items, each with 10 pairs (for a total of 20 stimuli) of More Similar Coordinates (i.e., two highly related coordinate concepts) and 10 pairs (for a total of 20 stimuli) of Less Similar Coordinates (i.e., two less related coordinate concepts) were created in the following manner. First, 224 pairs of coordinates judged to be highly similar by the experimenter were selected from 72 of the categories from the master list used in Experiment 1. These pairs formed the initial pool of More Similar Coordinates. Some of these items paired two typical category exemplars; others paired two atypical exemplars. One such item pairing two typical exemplars and one such item pairing two atypical exemplars from the same category were then chosen and a pair of Less Similar Coordinates formed by interchanging the first terms of the two More Similar Coordinate pairs. For instance, if the two More Similar Coordinate pairs were A-B and C-D, then the two Less Similar Coordinate pairs were A-D and C-B. A total of 268 Less Similar Coordinates were so formed. Semantic similarity ratings to the entire set of More Similar and Less Similar Coordinate items were then obtained from a group of 7 participants (separate from those who participated in the experiment proper). Ratings were on a scale from 1, representing very dissimilar in meaning, to 6, representing very

similar in meaning. All pairs of More Similar Coordinates and their corresponding pairs of Less Similar Coordinates were then retained for which both More Similar Coordinate items received a mean semantic similarity rating above 4.0 and both Less Similar Coordinate items received a mean semantic similarity rating below 4.0. In addition, for each pair of More Similar Coordinate items, one item had to pair two exemplars that were typical of the category from which they were drawn and the other had to pair two atypical exemplars. A total of 42 pairs of More Similar Coordinates and their corresponding 42 pairs of Less Similar Coordinates met these criteria. Two pairs of More Similar Coordinates and their corresponding Less Similar Coordinates were then randomly discarded. From the remaining stimuli, four lists of Coordinate false items were generated by randomly assigning 10 pairs of More Similar Coordinates to each list and then adding to each list the 10 pairs of Less Similar Coordinates derived from those More Similar Coordinates. Hence, each list consisted of 20 More Similar Coordinates and 20 corresponding Less Similar coordinates. These lists are shown in Appendix 2.

From each of the two true lists, two lists of Category-Derived Anomalous false items were created by randomly re-pairing the first and second terms of the true stimuli, with the restriction that no obvious relation exist between the two words of a re-paired item. Four lists of Coordinate-Derived Anomalous false items were similarly constructed from the four lists of Coordinate false items.

Associative Network Models of Lexical Semantic Memory

This section describes two associative network models of lexical semantic memory, one referred to as a sparse network model, the other as a dense network model. Both predict the equal-sized typicality effects across the Anomalous, Reversed, and Coordinate conditions of the experiments reported in the main body of the present paper as well as elsewhere (Gruenenfelder, 1986; McCloskey & Glucksberg, 1979), as well as the overall increase in latencies to true items in the Reversed and Coordinate conditions

relative to the Anomalous condition. Similarly, both models do not contain enough information to discriminate Cross-Category false items from true items in a category verification task. Hence, when such a discrimination is required, additional semantic processing that is outside the domain of the model must be invoked. In this sense, the models are compatible with, though do not strictly speaking predict, the increased typicality effect in the Cross-Category condition, for reasons explained in the main text. Neither model is complex—both are simple additive models. The reason for describing them in detail is simply to make as clear as possible the assumptions underlying the models.

Following existing network models (Collins & Quillan, 1969; Glass & Holyoak, 1975; Holyoak, 2008; Holyoak & Glass, 1975), the models represent words as nodes in a network connected by edges. More specifically, edges connect words that are associated with one another. Further, the edges are labeled to indicate the semantic relation between the pair of words connected by that edge. These relations are not limited to taxonomic relations but may cover a wide diversity of semantic relations—see McRae, Khalkhali, and Hare (2012), Santos, Chaigneau, Simmons, and Barsalou (2011), and Wu and Barasalou (2009) for taxonomies of such relations.

Words presumably become associated with one another through the standard principles of associative learning (e.g., Rescorla, 1988). In particular, words that frequently co-occur with one another relative to their total frequency of occurrence become associated (cf. Gruenenfelder, Recchia, Rubin, & Jones, 2016). The label—i.e., the particular semantic relation between those words—is determined by the syntactical structures in which they co-occur. A great deal of work has addressed how set—superset and coordinate relations can be determined in such a manner, beginning with the seminal work of Caraballo (1999) and Hearst (1992). Since then, that approach has been extended to include a wider variety of sematic relations (e.g., Baroni, Murphy, Barbu, & Poesio, 2010).

Associations between words vary in strength, that strength being determined by the relative frequency with which the two words co-occur. Stronger associations are retrieved more quickly than weaker associations.

A Sparse Associative Network Model

As mentioned, words become associated with one another because they co-occur with one another. That co-occurrence may be in writing, speech, or thought. In the sparse model, these associations are the only basis for forming an association. Because there exist more and larger corpora of written language than of spoken language and because corpora of thoughts simply do not exist, text corpora are usually used to derive an approximation of the network. In any event, and limiting ourselves to taxonomic relations for the time being, not all words that are coordinate to one another or that stand in a set—superset relation co-occur frequently enough with one another for an association to form between those words. Consequently, the network is *sparse*. Not all possible and true taxonomic relations are represented within that network (see also Shwartz, Goldberg, & Dagan, 2016). For that reason, using the network as a basis for responding in a category verification task requires relying on what might be called the near-transitivity of

taxonomic relations. If A is coordinate to B and B is coordinate to C, then A is coordinate to C. If A is coordinate to B and B is a subset of X, then A is may well be a subset of X (*Fig* can be considered coordinate to the vegetable *okra*. Nevertheless, *fig* is a *fruit*, not a *vegetable*.) Whether a person is willing to infer, in the context of a category verification task, whether A being coordinate to B and B being a subset of X means that A is also a subset of X, depends upon the semantic relation(s) holding between the two terms in false stimuli, as explained in the main text. In a context of Coordinate false items, such an inference would be reasonable. In a context of Cross-Category false items ("A fig is a vegetable."), such an inference would not be justified.

Some notation is introduced in order to make further explication of the model more concise. The symbols $\subset$, $\supset$, and $\equiv$ are used to indicate subset, superset, and coordinate relations, respectively. Hence $X \subset Y$ means that X is a member of the set Y, $X \supset Y$ that the set X contains the member Y, and $X \equiv Y$ that X and Y are coordinates (or hyponyms). Retrieval in the network consists of traversing edges first from the node (word) from which the retrieval process is initiated, then traversing edges from those retrieved nodes to additional nodes to which they are directly connected, then traversing edges from those additionally retrieved nodes to the nodes to which they are directly connected, and so on. Nodes A and B are directly connected if there is an edge between A and B. They are indirectly connected if there is no such edge but there is a path from nodes A to B going through one or more intermediate nodes. People are assumed to be able to choose which edges to traverse based on the label on that edge, i.e., the semantic relation it represents. In the category verification task, only edges with $\subset$, $\supset$, and $\equiv$ labels are assumed to be traversed. All others are ignored. For purposes of the present experiments, this assumption is not critical. It could also be assumed that all edges are traversed (retrieved), but only those exhibiting the $\subset$, $\supset$, and $\equiv$ are kept for further processing.

In a category verification task, given the stimulus, "An S is a P," (abbreviated as S—P) two retrieval processes are initiated and executed in parallel, one from the node corresponding to S and one from the node corresponding to P. The retrieval process continues until either 1) the P node is retrieved by the retrieval process initiated from the S node (either directly or via a chain of associations), 2) the S node is retrieved by the retrieval process initiated from the P node, or 3) a threshold path length is reached. Here, that threshold path length, as indicated in the main text, is assumed to be 2. In other words, for the retrieval process initiated from the S node, all directly connected nodes are first retrieved. If those nodes do not include P, then all nodes directly connected to those nodes are also retrieved. The retrieval process then ends, even if the node P has still not been retrieved.

The term *retrieval race* or *winning the retrieval race* is used to indicate that whichever of the multiple paths that connect the S and P terms is the first to be retrieved determines the response that is made.

The reason the threshold path length is set to a perhaps seemingly low value of 2 is simply because networks built from text corpora have a relatively small mean shortest path length between any two arbitrary nodes. It tends to be only slightly greater than 3 (e.g., Gruenenfelder et al., 2016). In other words, any two arbitrarily selected nodes from

such a network can be reached by traversing just 3 edges, suggesting that words that are semantically related to one another are connected by paths consisting of fewer than 3 edges. Note that this mean shortest path length can only get shorter if we add to the network edges due to additional co-occurrences in spoken language.

In the Anomalous condition, where all false stimuli pair two semantically unrelated words, the retrieval process for true stimuli always (or nearly always) terminates because condition (1) or (2) above is met—that is, an association or chain of two associations linking S and P are retrieved—whereas for false stimuli the retrieval process terminates because condition (3) above is met—no association or chain of two associations linking S and P in false stimuli is retrieved. Hence, a participant can safely make a true response if an association or chain of two associations is retrieved, and a false response if no such chain can be retrieved. Hence the total time to respond consists of the time to retrieve the association or associative chain, *r*, plus the time to read the stimulus and execute the response, *c*. Although the term *c* may differ for true and false stimuli (It may take different amounts of time to execute true and false responses), it is not assumed to differ between true stimuli containing typical category exemplars and true stimuli containing atypical category exemplars. Indeed, the results of Experiment 2 of the present paper provide some direct support for that later assumption. Retrieval time, *r*, on the other hand is assumed to be different for typical and atypical true stimuli, since atypical stimuli involve retrieving a weaker association or are more likely to involve retrieving a chain of two associations rather than a single direct association. In other words, $r_t < r_a$, where r_t is retrieval time for typical exemplars and r_a is retrieval time for atypical exemplars. Letting $\mathrm{RT_t}$ and $\mathrm{RT_a}$ be mean reaction times to typical and atypical exemplars, respectively, gives

$$\mathrm{RT_t} = c + r_t < \mathrm{RT_a} = c + r_a$$

The difference between $\mathrm{RT_t}$ and $\mathrm{RT_a}$ is, of course, the typicality effect.

In the Reversed and Coordinate conditions, all false stimuli (as well as true stimuli) pair semantically related words. Therefore, for both true and false stimuli, the retrieval process always (or nearly always) terminates because condition (1) or (2) above is met, and never (or extremely rarely) terminates because condition (3) above is met. That is, it never terminates due to a failure to retrieve an association or a chain of two associations linking S and P. Consequently, the fact that P was retrieved from S, or S was retrieved from P, is not a sufficient basis for responding. In this case, an additional process, referred to here as path evaluation, must follow the retrieval process in order to determine the truth or falsity of the stimulus.

The path evaluation process consists of indexing into a set of production rules with the labels on the retrieved path of associations linking the two terms of the stimulus. Since the retrieval process terminates after searching paths up to and including a length of two, that index is simply a string of two symbols. In the case of a direct association between the two terms, the second "association" in the path is indicated by the null symbol $\varnothing$. The complete set of production rules for the Coordinate and Reversed conditions is shown and discussed later.

It is not necessarily the case that, for true stimuli, the proportion of typical exemplars that would result in a given rule firing would equal the proportion of atypical

exemplars firing that rule. That is, one rule might tend to be activated more by typical exemplars and another rule more by atypical exemplars. For example, a direct association is probably more likely to win the retrieval race for typical than atypical exemplars. However, there appears to be no motivated reason to think that any one rule is favored over any other rule. In other words, on average, the time to activate or fire a rule is the same across all the rules. That time is referred to here as p. For reasons discussed below, p may differ across the Coordinate and Reversed conditions. Hence p_c is the time to activate a production rule in the Coordinate condition and p_r is the time to activate a production rule in the Reversed condition. It is this need to activate a production rule that differentiates the Coordinate and Reversed conditions, on the one hand, from the Anomalous condition, on the other. Assuming that the relation must be retrieved before it can activate a production rule, the time p must be added to the equation for RT for the Anomalous condition in order to determine the RT for the Coordinate and Reversed conditions. In the Coordinate condition, reaction time to typical true exemplars becomes

$$\mathrm{RT_t} = c + r_t + p_c$$

And reaction time to atypical true exemplars becomes

$$\mathrm{RT_a} = c + r_a + p_c$$

In words, reaction times to true stimuli increase in the Coordinate condition relative to the Anomalous condition, but by the same amount to typical and atypical stimuli.

Similarly, in the Reversed condition, reaction time to typical true exemplars becomes

$$\mathrm{RT_t} = c + r_t + p_r$$

And reaction time to atypical true exemplars becomes

$$\mathrm{RT_a} = c + r_a + p_r$$

In words, reaction times to true stimuli increase in the Reversed condition relative to the Anomalous condition, but by the same amount to typical and atypical stimuli.

Table NS-1, below, shows the set of production rules—i.e., the mapping from the indices to the appropriate response—for the search commencing with the S term in the Reversed condition. Table NS-2 shows the production rules for the search commencing with the P term in the Reversed condition. Analogously, Tables NS-3 and NS-4 show the production rules for the searches commencing with the S and P terms, respectively, in the Coordinate condition. For each rule, the table also gives an example, for an illustrative stimulus, of the chain of retrieved associations that would trigger that rule. Note that the mapping of a retrieved chain to a response depends upon the specific false stimuli being used. The chain $\equiv \subset$, for instance, in the Coordinate condition would indicate a true stimulus, but that same chain would be ambiguous in the Cross-Category condition. (In contrast, the chain $\equiv \equiv$ would always indicate a coordinate relation between the two terms.

Some retrieved associative chains, in particular a subset relation followed by a superset relation (⊂ ⊃) or a superset relation followed by a subset relation (⊃ ⊂) can be ambiguous. Such chains are so marked in the table below. Ambiguous cases are discussed in more detail below.

Also, again depending upon False condition and upon whether the search commences with the subject term or the predicate term, some associative chains simply cannot be retrieved. For example, in the Coordinate condition, it is not possible to retrieve from the S term of a stimulus a chain of two superordinate relations (⊃ ⊃) terminating at the P term. Such impossible cases are also noted in the tables below. Similarly, in the Reversed condition, starting at either term, it is not possible to retrieve a chain of two coordinate relations (≡ ≡) terminating at the other term. Such cases are marked in the tables as "Would not occur."

Given three possible relations (⊂, ⊃, and ≡) there are 12 possible production rules for each search (including those described above that in fact would never occur).

Table NS-1. Production rules mapping the chain of retrieved relations to a true or false response for the Reversed condition for the search that begins with the S term of the stimulus.

Chain of Associations	Response	Example (for True Stimulus Car—Vehicle
⊂ ⊂	True	Car—Motorized_Vehicle—Vehicle
≡ ⊂	True	Car—Truck—Vehicle
⊂ ≡	True	Car—Tool—Vehicle<br>The example is a bit contrived, but consider Fig—Vegetable—Fruit for the stimulus Fig—Fruit
⊂ ∅	True	Car—Vehicle
		Example (for False Stimulus Vehicle—Car)
⊃ ⊃	False	Vehicle—Motorized_Vehicle—Car
⊃ ≡	False	Vehicle—Truck—Car
≡ ⊃	False	Vehicle—Tool—Car
⊃ ∅	False	Vehicle—Car
⊃ ⊂	Ambiguous	For true stimulus, mammal—animal<br>retrieved path of mammal->cat->animal<br>For false stimulus, animal—mammal<br>retrieved path of animal->cat->mammal
⊂ ⊃	Ambiguous	For true stimulus, cat—mammal<br>retrieved path of cat->animal->mammal<br>For false stimulus, mammal—cat<br>Retrieved path, mammal->animal->cat
≡ ≡	Would not occur	

≡ ∅	Would not occur	

Table NS-2. Production rules mapping the chain of retrieved relations to a true or false response for the Reversed condition for the search that begins with the P term of the stimulus.

Chain of Associations	Response	Example (for False Stimulus Vehicle—Car
⊂ ⊂	False	Car—Motorized_Vehicle—Vehicle
≡ ⊂	False	Car—Truck—Vehicle
⊂ ≡	False	Car—Tool—Vehicle<br>The example is a bit contrived, but consider FIg—Vegetable—Fruit for the stimulus Fig—Fruit
⊂ ∅	False	Car—Vehicle
		Example (for FTrue Stimulus Car—Vehicle)
⊃ ⊃	True	Vehicle—Motorized_Vehicle—Car
⊃ ≡	True	Vehicle—Truck—Car
≡ ⊃	True	Vehicle—Tool—Car
⊃ ∅	True	Vehicle—Car
⊃ ⊂	Ambiguous	For true stimulus, cat—mammal<br>retrieved path of mammal->Calico->cat<br>For false stimulus, mammal—cat<br>Retrieved path, cat->Calico->mammal
⊂ ⊃	Ambiguous	For true stimulus, cat—mammal<br>retrieved path of mammal->animal->cat<br>For false stimulus, mammal—cat<br>Retrieved path, cat->animal->mammal
≡ ≡	Would not occur	
≡ ∅	Would not occur	

Table NS-3. Production rules mapping the chain of retrieved relations to a true or false response for the Coordinate condition for the search that begins with the S term of the stimulus.

Chain of Associations	Response	Example (for True Stimulus Car—Vehicle
⊂ ⊂	True	Car—Motorized_Vehicle—Vehicle
≡ ⊂	True	Car—Truck—Vehicle
⊂ ≡	True	Car—Tool—Vehicle<br>The example is a bit contrived, but consider Apple—Vegetable—Fruit for the stimulus Apple—Fruit
⊂ ∅	True	Car—Vehicle

		Example (for False Stimulus Car—Truck)
≡ ≡	False	Car—Bus—Truck
≡ ∅	False	Car—Truck
⊃ ⊂	Ambiguous/true?	For true stimulus, mammal—animal<br>Retrieved path mammal—cat—animal<br>For false stimulus, fruit—vegetable<br>Retrieved path fruit—tomato--vegetable
⊂ ⊃	Ambiguous	For true stimulus, cat—mammal<br>Retrieved path cat—animal—mammal<br>False stimulus, cat—dog<br>Retrieved path cat—animal--dog
⊃ ⊃	Would not occur	
⊃ ≡	Would not occur	
⊃ ∅	Would not occur	
≡ ⊃	Would not occur	

Table NS-4. Production rules mapping the chain of retrieved relations to a true or false response for the Coordinate condition for the search that begins with the P term of the stimulus.

Chain of Associations	Response	Example (for True Stimulus Car—Vehicle
⊃ ⊃	True	Vehicle—Motorized_Vehicle—Car
≡ ⊃	True	Vehicle—Tool—Car
⊃ ≡	True	Vehicle—Truck—Car
⊃ ∅	True	Vehicle—Car
		Example (for False Stimulus Car—Truck)
≡ ≡	False	Truck—Bus—Car
≡ ∅	False	Truck—Car
⊃ ⊂	Ambiguous/true?	For true stimulus, cat—mammal<br>Retrieved path mammal—dog—cat<br>For false stimulus, fruit—vegetable<br>Retrieved path vegetable—tomato—fruit
⊂ ⊃	Ambiguous	For true stimulus, cat—mammal<br>Retrieved path mammal—animal—cat<br>For false stimulus, cat—dog<br>Retrieved path cat—animal—dog
⊂ ⊂	Would not occur	
⊂ ≡	Would not occur	
⊂ ∅	Would not occur	
≡ ⊂	Would not occur	

Ambiguous Paths

As noted in Tables NS-1 through NS-4, some paths of retrieved relations can be ambiguous given the discrimination required in a particular False Condition. For example, in the Reversed condition, consider the true stimulus “cat—mammal.” It is possible, starting with the S term, to retrieve a path of (two) associations from cat to mammal by first retrieving a subset relation from cat to animal and then a superset relation from animal to mammal. But now consider the false stimulus “mammal—cat.” Here, again starting with the S term, a path of two associations consisting of a subset relation followed by a superset relation can also be retrieved: mammal to animal and then animal to cat. Hence the retrieval index $\subset \supset$ is ambiguous—it can occur for both true and false stimuli. Similarly, the index $\supset \subset$ is ambiguous. Consider the same true stimulus as above, cat—mammal, and the retrieval process originating with the S term. A superset relation could first be retrieved from cat to Calico and then a subset relation from Calico to mammal. And for the false stimulus, mammal—cat, and the retrieval process beginning with the S term, the superset relation from mammal to Calico could first be retrieved followed by the subset relation from Calico to cat.

An important fact about these ambiguous retrieval paths to keep in mind is that in the Anomalous condition, they are not at all ambiguous. In that condition, a true decision can be relatively safely made if a path of length 1 or 2 linking the subject and predicate terms can be retrieved. The labels on those retrieved paths are of no consequence. Hence, if the ambiguous paths fairly frequently win the retrieval race (i.e., are the first path retrieved of all the possible paths between the S and P terms), and if resolving the ambiguity lengthens the retrieval process, and if the frequency of occurrence of the ambiguous cases (not the number of possible ambiguous cases, but the frequency with which an ambiguous chain wins the retrieval race) is different for typical and atypical true exemplars, than the prediction of the model of equal-sized typicality effects in the Coordinate and Reversed condition as in the Anomalous condition is weakened.

Faced with the situation where an ambiguous path wins the retrieval race, what are a participant’s options? There seem to be three reasonable options. First, although the model as originally stated stops the retrieval process once a single path linking the S and P terms has been retrieved, there is no theoretical reason why the retrieval process could not continue. In the event of the first retrieved path being ambiguous, the second retrieved path is used to index into the production rules. Hence, total retrieval time will be longer for those cases where an ambiguous path is retrieved. If the likelihood of retrieving an ambiguous path differs for typical and atypical true exemplars, the typicality effect could change in the Coordinate or Reversed condition relative to the Anomalous condition due to this increased retrieval time. However, this increase in retrieval time is likely to be very small and hence extremely hard to detect. First, the increase only occurs on trials on which an ambiguous path wins the retrieval race, and such trials themselves are likely to be rare (see below). Second, when the increase does occur, the size of the increase is likely to be small. It is simply the time to retrieve the second path minus the time to retrieve the first path. A small effect that occurs on a small proportion of trials will be hard to detect when examining mean reaction times across a number of trials. Remember also that for these increased retrieval times to be reflected in a change in the

size of the typicality effect, ambiguous paths need to win the retrieval race much more frequently for typical exemplars than for atypical exemplars, or vice versa.

A second possible strategy that participants may use to deal with retrieval of ambiguous cases is simply to guess at the answer. Given that the task is a speeded task, such a strategy is not unreasonable, even though the ambiguous cases could be disambiguated by continuing the retrieval process until an unambiguous path is retrieved. Such a strategy would increase the error rate but so long as an ambiguous path winning the retrieval race is a rare event, the increase may well be within what the participant judges to be a reasonable amount. Theoretically, the time to make the guess could be the same as, less than, or greater than the time to execute a production rule. If it is the same (or nearly the same), then the typicality effect would also be the same in the Coordinate and Reversed conditions as in the Anomalous condition. If it is less than or greater than the time to execute a production rule, the typicality effect could change, but only if an ambiguous path winning the retrieval race is a relatively frequent event, and its relative frequency is different for typical and atypical exemplars.

A third possible strategy that participants may use to deal with retrieval of ambiguous cases is simply to assign either a true or a false response to such cases in the set of production rules. For instance, perhaps a participant believes that retrieving a $\supset \subset$ path in the Coordinate condition is more likely to indicate a subset—superset relation than a coordinate relation. In that case, the participant may simply set the production rule to indicate a true response in the event of retrieving such a path. With this strategy, there is no change in retrieval times for either typical or atypical exemplars in the Coordinate or Reversed conditions relative to the Anomalous condition (though error rates could be [slightly] affected). Likewise, because the ambiguous paths lead to a production rule just like any other retrieved path, path evaluation times would not be differentially affected for typical and atypical exemplars. Hence, this strategy would lead to the same prediction as the model without ambiguous paths—the size of the typicality effect would be the same in the Coordinate and Reversed conditions as in the Anomalous condition.

Although the third strategy for handling the retrieval of ambiguous paths does not affect the size of the typicality effect (as measured by reaction times), the first two strategies (1) continuing the retrieval process, or 2) guessing could affect the typicality effect *provided that three additional conditions are met*. First, for both strategies, the ambiguous path needs to win the retrieval race a significant number of times. Second, for both strategies, the number of times that the ambiguous path wins the retrieval race has to differ significantly between typical and atypical true exemplars. Third, in the case of the first strategy, that is, the strategy of continuing the retrieval process, on those trials where the ambiguous path does win the retrieval race, the time to retrieve the runner-up, non-ambigous path needs to be significantly longer than the time to retrieve the winning ambiguous path. (Hopefully it is obvious that the word “significant” is being used in this paragraph in the sense of “important,” not in the sense of “statistically significant.”)

As an example, let’s consider the first strategy: in the event that an ambiguous path is retrieved, the retrieval process continues until an unambiguous path is retrieved (which is likely to be the second retrieved path, as there are only so many ambiguous paths, but many unambiguous paths, linking the subject and predicate terms). Suppose that on 0% of the trials involving typical exemplars, an ambiguous path is retrieved and

that on 5% of the trials involving an atypical exemplar, an ambiguous path is retrieved (These estimates are likely high—see below). Suppose further that continuing the retrieval process adds 20 ms to overall reaction times. Under these assumptions, relative to the Anomalous condition, no time is added to retrieval time for typical exemplars. For atypical exemplars, mean retrieval time increases by .05 * 20 ms = 1 ms. Therefore, the increase in the typicality effect is 1 – 0 = 1 ms (More generally, this situation applies to the case where the ambiguous path is retrieved 5% more frequently for atypical than for typical exemplars.). Similarly, if an ambiguous path is retrieved 10% more frequently for atypical than typical exemplars, then the increase in the typicality effect is 2 ms. If the differential is 20%, the effect is still only 4 ms. Even with a differential of 50% in the frequency with which an ambiguous path is retrieved for typical and atypical exemplars, the increase in the size of the typicality effect is just 10 ms. This last effect is perhaps large enough to be detectable; the first three effects would be extremely difficult to detect.

Similar comments apply to the guessing strategy. The difference in the frequency with which an ambiguous path is retrieved for typical and atypical exemplars and the increased time to make the guess are both large, then any change in the size of the typicality effect would be difficult to detect.

The above arguments suggest that if retrieval of ambiguous paths has any effect on the size of the typicality effect, it is quite small. The argument depends on the frequency with which an ambiguous path is retrieved being substantially different for typical and atypical exemplars. The suggestion here is that that difference has to be small, because the absolute frequency with which the ambiguous path is retrieved is likely to be small. There simply seems to be many more unambiguous paths linking the S and P terms than ambiguous paths, making it less likely that the ambiguous path wins the retrieval race. For example, consider the true stimulus from above, cat—mammal and the $\subset \supset$ path, i.e., a path consisting of a subset relation followed by a superset relation, e.g. cat->animal->mammal. That seems to be one of at best just a few paths involving a $\subset \supset$ chain of associations linking cat and mammal. (True, there may be a path from cat to living thing to mammal, but how strong are the associations in that path?) That path does seem to contain associations that are perhaps moderately strong (cat to animal and animal to mammal). The other ambiguous chain, $\supset\ \subset$, a superset relation followed by a subset relation seems to be even less likely to win the retrieval race. There are perhaps more paths in this chain (cat to calico, cat to tabby, cat to Siamese and then any of those terms to mammal), but how strong is the association from tabby to mammal, or calico to mammal, or tabby to mammal? These paths are competing against the (potential) direct path from cat to mammal, as well as all those paths from cat to a coordinate of cat to mammal. Many of these paths would seem to be as strong as or stronger than the cat to animal to mammal path, making it unlikely that the latter wins the retrieval race with any significant frequency. Hence, the likelihood that the difference in the frequency with which ambiguous paths are retrieved for typical and atypical exemplars is unlikely to be as high as 10%, much less 20%, and certainly not 50%.

Cat—mammal was not an actual stimulus in the experiments reported in the main body of the paper. For some of the actual stimuli, as in the case for cat—mammal, it may not be unreasonable to assume that a $\subset \supset$ path occasionally wins the retrieval race. For

instance, for the stimulus lion—carnivore, perhaps the path lion—animal—carnivore is an occasional winner. On the other hand, for many stimuli, it seems very unlikely that such a path would ever win the retrieval race. Robbery—crime is one such stimulus. Couch—furniture is another. Is couch—object—furniture likely to win the retrieval race? Similar considerations apply to the $\supset$ $\subset$ path. Considerations such as these reinforce the conclusion that the difference in the frequency with which ambiguous paths are retrieved for typical and atypical exemplars is very unlikely to have a detectable effect on the size of the typicality effect, and hence that the prediction of equal-size typicality effects in the Reversed and Coordinate conditions as in the Anomalous condition is valid.

Path Evaluation in the Coordinate versus Reversed Condition

The results of Experiment 1 indicate that the path evaluation process for true items in the Reversed condition is much longer than in the Coordinate condition. Given that in each case, path evaluation involves indexing into a table of production rules, why might this difference occur? An inspection of Tables NS-1 through NS-4 suggests a reason. In the Coordinate condition, the same response is made to a given index, regardless of whether it was retrieved from the S term of the stimulus or the P term. Hence, there is no need to track whether the index resulted from the search beginning with the S term or the search beginning with the P term. In the Reversed condition, the situation is quite a bit different. Here, where the response is "true" for an index retrieved from the S term, it is "false" for the same index retrieved from the P term, and vice versa. Hence, it is critical to track whether the index resulted from the retrieval process originating from the S term or the retrieval process originating from the P term. This additional cognitive load may result in the larger increase in response times to true items in the Reversed condition than in the Coordinate condition.

Cross-Category Condition

If ambiguous paths are so easily handled in the Coordinate and Reversed conditions, why are they not just as easily handled in the Cross-Category condition, and why then is an increased typicality effect observed in that condition? The answer is because all (or at least nearly all) paths retrieved in this condition are ambiguous. They are ambiguous because coordinate relations can cross category boundaries. Because apple can co-occur with carrot in the syntactical structures that indicate a coordinate relation, there is every reason to believe that a coordinate relation can be formed between apple and carrot, a coordinate relation that crosses the vegetable—fruit category boundary. It is even possible for apple to form direct subset links with both vegetable and fruit. And coordinate associations do cross category boundaries. Consider the coordinate pair robin—cardinal. Suppose a person carrying that coordinate association around in their head now develops some expertise with birds, and learns that robins are a member of the class of thrush and cardinals are a member of the class of finches (coordinate to thrushes). Does that person now break the coordinate relation between robin—cardinal or does that relation remain, a relation that crosses the thrush—finch category boundary? Given that all retrieved paths in the Cross-Category condition are ambiguous, *given the discrimination between true and false stimuli required in that condition*, some strategy other than reliance on retrieving and evaluating simple paths in an associative network is necessary to support performance in the condition.

Falsifying the Model

Especially in lieu of the above discussion on ambiguous paths, some people might feel that the sparse network model has become non-falsifiable. If the typicality effect does not change across two conditions, it is because very few paths are ambiguous. If it increases, it is because ambiguous paths are much more likely for atypical than typical exemplars. If it decreases, it is because ambiguous paths are much more likely for typical than atypical exemplars. Although I am sure that other researchers can be much more creative in determining ways of falsifying the model, some approaches are suggested here.

First, if an increased typicality effect is observed in a condition where the model would seem to predict no change in the effect, and a claim is made that the increase is due to an extraordinary large number of ambiguous paths are retrieved in the case of atypical exemplars, some form of motivated argument has to be made concerning why in that condition the frequency of ambiguous paths winning the retrieval race is apparently so much higher than in the category verification task used here.

Second, the present study examined only set—superset and coordinate relations. A variety of other relations can also be determined via the syntactical structures in which words co-occur (Baroni et al., 2010). Many such relations would seem to be discriminable from, for example, set—superset relations within an associative network. Further, the discrimination seems to take the form of simply indexing into a set of production rules with the retrieved relations, just as in the present case of discriminating set—superset relations from coordinate relations. Hence, just as the typicality effect is invariant across the Anomalous, Reversed, and Coordinate conditions, we would expect it to be invariant across an Anomalous condition and conditions requiring discrimination set—superset relations from these other relations.

There is, of course, no reason to focus exclusively on the typicality effect in set—superset relations. Some coordinate stimuli pair words more strongly related to one another than to other coordinate stimuli. We would expect this "coordinate similarity" effect to also be invariant across conditions requiring discrimination of coordinate stimuli from anomalous stimuli, coordinate stimuli from set—superset stimuli, and coordinate stimuli from various other semantic relations, per the discussion of the typicality effect immediately above. In conducting and interpreting such experiments, it is important to keep in mind the polarity-correspondence principle (Proctor & Cho, 2006; Proctor & Xiong, 2015) and not compare response times to coordinate stimuli in one condition where a true response is made to them to response times to coordinate stimuli in another condition where a false response is made to them.

A Dense Associative Network Model

There are ways of taking a sparse associative network, like the one described above, and turning it into a denser associative network, in which the semantic relation existing between more and more pairs of words is directly represented in the network. Using distributional information, sometimes combined with syntactical information, various algorithms have had decent success discrimination which of two semantic relations exists between two words (Baroni, Bernardi, Do, & Shan, 2012; Nguyen, Köper, Schulte im Walde, & Vu, 2017; Nguyen, Schulte im Walde, & Vu, 2016; Roller, Erk, & Boleda, 2014;

Shwartz et al., 2016; Weeds, Clarke, Reffin, Weir, & Keller, 2014). Such work suggests that it may be possible to determine the semantic relation that exists between pairs of words that do not co-occur with one another based on other words that they do co-occur with in common, and the syntactical patterns involved in those co-occurrences. If humans apply such algorithms to their own lexical semantic memory networks, and then directly encode the inferred relation, a denser network would result.

The network would also become denser if, when people make inferences based on the network, the results of those inferences were directly stored. For example, knowing that A is a subset of B and B of C allows the inference that A is a subset of C. Knowing that A is a subset of B and B has property C allows the inference that A has property C. Knowing that A is a coordinate of B and B of C allows the inference that A is also a coordinate of C. If when such inferences are made, the result is stored as part of the network, the network would also become denser.

If the network becomes dense enough, then, when performing tasks such as category verification, the retrieval process can stop after retrieving all paths of just length 1 rather than retrieving all paths of length 1 or 2 before terminating. Retrieval time differences between pairs of words would still exist due to differences in relation strength. However, all retrieved paths would be of length 1. In terms of the present experiment, they would all indicate either a set—superset, a superset—set, or a coordinate relation. In terms of discriminating Reversed or Coordinate false stimuli from true stimuli in category verification, there would be no ambiguous paths in this denser network. However, to the extent that the algorithms for determining set—superset relations are less than perfect, set—superset relations may be inferred from word A to B, but also from A to C, where C is a coordinate of B. In such a case, ambiguity would still exist in the case of Cross-Category false stimuli, forcing reliance on some strategy other than simple retrieval plus path evaluation from a semantic network.

From Where Does the Network Originate?

Where does the network come from? Words presumably become associated with one another via normal processes of associative learning (Rescorla, 1988) because they co-occur with one another in either spoken or written speech. Hence, an at least rough approximation of the network can be gleaned by examining the pattern of co-occurrences in an appropriate corpus (cf. Gruenenfelder et al., 2016). Furthermore, Caraballo (1999) and Hearst (1992) have shown that the syntactical structures in which coordinate terms co-occur can also be used to identify the relation between those terms as being one of coordination, as opposed to some other semantic relation, such as “isa” or “has” (see alsoFrermann & Lapata, 2016; Han et al., 2015; Huang, Wan, & Xiao, 2015; Manning, 1993; Nguyen, Schulte im Walde, & Vu, 2017; Ohshima, Oyama, & Tanaka, 2006; Riloff & Shepherd, 1997; Sanderson & Croft, 1999; Shinzato & Torisawa, 2004; Snow, Jurafsky, & Ng, 2005). Baroni et al. (2010) have generalized this approach of examining the syntactical structures in which words co-occur to algorithmically determine word pairs that share other semantic relations, such has *has a, found in*, and *part of* [see Baroni et al. (2010) for additional examples; see also Girju, Badulescu, and Moldovan (2006)]. Such results suggest that the language environment has sufficient information for people to form semantic networks containing these and perhaps other relations.

Distributional Models as Feature Models

As described in the main paper, distributional or high dimensional spatial models of semantic memory represent the meaning of a concept as a point in a high-dimensional space (Louwerse, Cai, Hu, Ventura, & Jeuniaux, 2006). That point can be described as a vector where each element of the vector is the value that concept possesses on the corresponding dimension of the space. These models tend to be scant on processing assumptions, and have rarely been applied to explaining performance in the category verification task-- is an exception. To the extent that the dimensions can be thought of as features, however, these models are quite similar to feature models. Like feature models, they also emphasize semantic similarity. In these spatial models, the semantic similarity of two concepts is defined as the cosine of the angle between the two vectors representing those concepts. However, even in the Anomalous condition, it does not appear as if a simple computation of the cosine is what underlies the typicality effect. Why does calculating a cosine farther from 1 take longer than computing a cosine near 1? Instead of computing cosine similarity, perhaps people compare the terms' values on the various dimensions in the space to determine category membership. This approach is quite like the feature comparison process in feature models. The values on the dimensions are in a sense features. For these reasons, high-dimensional spatial models are treated here together with feature models, and share with those models the ease of explaining the increased typicality effect in the Cross-Category condition and the difficulty of explaining the equal sized typicality effects across the Anomalous, Coordinate, and Reversed conditions.

References

Baroni, M., Bernardi, R., Do, N.-Q., & Shan, C.-c. (2012). Entailment above the word level in distributional semantics. *Proceedings of the 13th Conference of the European Chapter of the Association for Computational Linguistics*, 23-32.

Baroni, M., Murphy, B., Barbu, E., & Poesio, M. (2010). Strudel: A corpus-based semantic model based on properties and types. *Cognitive Science, 34*, 222-254. doi:10.1111/j.1551-6709.2009.01068.x

Caraballo, S. A. (1999). Automatic construction of a hypernym-labeled noun hierarchy from text. In *Proceedings of the 37th Annual Meeting of the Association for Computational Linguistics (ACL-1999)* (pp. 120-126). College Park, MD: Association for Computational Linguistics.

Collins, A. M., & Quillan, M. R. (1969). Retrieval time from semantic memory. *Journal of Verbal Learning and Verbal Behavior, 8*, 240-247.

Frermann, L., & Lapata, M. (2016). Incremental Bayesian category learning from natural language. *Cognitive Science, 40*, 1333-1381. doi:10.1111/cogs.12304

Girju, R., Badulescu, A., & Moldovan, D. (2006). Automatic discovery of part-whole relationships. *Computational Linguistics, 32*, 83-135.

Glass, A. L., & Holyoak, K. J. (1975). Alternative conceptions of semantic theory. . *Cognition, 3*, 313-339.

Gruenenfelder, T. M. (1986). Relational similarity and context effects in category verification. *Journal of Experimental Psychology: Learning, Memory, and Cognition, 12*, 587-599.

Gruenenfelder, T. M., Recchia, G., Rubin, T., & Jones, M. N. (2016). Graph-theoretic properties of networks based on word association norms: Implications for models of lexical semantic memory. *Cognitive Science, 40*, 1460 - 1495.

Han, H., Zhu, L., Zhang, Z., Li, L., Shi, C., & Gui, J. (2015). Extracting hierarchical relationship of scientific and technical terms from unstructured text. *Journal of Information & Computational Science, 12*, 5235 - 5245.

Hearst, M. A. (1992). Automatic acquisition of hypnonyms from large text corpora. In A. Zampolli (Ed.), *Proceedings of the International Conference on Computational Linguistics* (pp. 539-545). Nantes, France: COLING-92.

Holyoak, K. J. (2008). Relations in semantic memory: Still puzzling after all these years. In A. Lesgold, B. Ross, E. A. Loftus, & W. K. Estes (Eds.), *Memory and mind: A Festschrift for Gordon H. Bower.* (pp. 141-158.). New York: Lawrence Erlbaum Associates.

Holyoak, K. J., & Glass, A. L. (1975). The role of contradictions and counterexamples in the rejection of false sentences. *Journal of Verbal Learning and Verbal Behavior, 14*, 215-239.

Huang, X., Wan, X., & Xiao, J. (2015). Learning to mine Chinese coordinate terms using the web. *arXiv preprint arXiv:1507.02145*.

Louwerse, M., Cai, Z., Hu, X., Ventura, M., & Jeuniaux, P. (2006). Cognitively-inspired NLP-based knowledge representations: Further explorations of Latent Semantic Analysis. *International Journal on Artificial Intelligence Tools, 15*, 1021-1039.

Manning, C. D. (1993). Automatic acquisition of a large subcategorization dictionary from corpora. In *Proceedings of the 31st Annual Meeting of the Association for Computational Linguistics*. Stroudsburg, PA: Association for Computational Linguistics.

McCloskey, M., & Glucksberg, S. (1979). Decision processes in verifying category membership statements: Implications for models of semantic memory. . *Cognitive Psychology, 11*, 1-37.

McRae, K., Khalkhali, S., & Hare, M. (2012). Semantic and associative relations: Examining a tenuous dichotomy. In V. F. Reyna, S. Chapman, M. Dougherty, & J. Confrey (Eds.), *The adolescent brain: Learning, reasoning, and decision making* (pp. 39-66). Washington, D. C.: American Psychological Association.

Nguyen, K. A., Köper, M., Schulte im Walde, S., & Vu, N. T. (2017). Hierarchical embeddings for hypernynmy detection and directionality. *arXiv:1707.07273v1 [cs.CL]*.

Nguyen, K. A., Schulte im Walde, S., & Vu, N. T. (2016). Integrating distribution lexical contrast into word embeddings for antonym-synonym distinction. *arXiv:1605.07766v1 [cs.CL]*.

Nguyen, K. A., Schulte im Walde, S., & Vu, N. T. (2017). Distinguishing antonyms and synonyms in a pattern-based neural network. *arXiv:1701.02962v1 [cs.CL]*.

Ohshima, H., Oyama, S., & Tanaka, K. (2006). Searching coordinate terms with their context from the web. In K. Aberer, Z. Peng, E. A. Rundensteiner, Y. Zhang, & X. Li (Eds.), *Proceedings of the 7th International Conference on Web Information Systems Engineering* (pp. 40-47). Wuhan, China: Springer.

Proctor, R. W., & Cho, Y. S. (2006). Polarity correspondence: A general principle for performance of speeded binary classification tasks. *Psychological Bulletin, 132*, 416 - 442.

Proctor, R. W., & Xiong, A. (2015). Polarity correspondence as a general compatibility principle. *Current Directions in Psychological Science, 24*, 446 - 451. doi:10.1177/0963721415607305

Rescorla, R. A. (1988). Pavlovian conditioning: It's not what you think it is. *American Psychologist, 43*, 151-160.

Riloff, E., & Shepherd, J. (1997). A corpus-based approach for building semantic lexicons. *Proceedings of the Second Conference on Empirical Methods in Natural Language Processing*, http://www.aclweb.org/anthology/W/W97/W97-0313.pdf. Retrieved from http://www.aclweb.org/anthology/W/W97/W97-0313.pdf

Roller, S., Erk, K., & Boleda, G. (2014). Inclusive yet selective: Supervised distributional hypernymy detection. *Proceedings of COLING 2014, the 25th International Conference on Computational Linguistics: Technical Papers*, 1025-1036.

Sanderson, M., & Croft, B. (1999). Deriving concept hierarchies from text. In F. Gey, M. Hearts, & R. Tong (Eds.), *Proceedings of the 22nd Annual International ACM SIGIR Conference on Research and Development in Information Retrieval* (pp. 206-213). Berkeley, CA: ACM.

Santos, A., Chaigneau, S. E., Simmons, W. K., & Barsalou, L. (2011). Property generation reflects word association and situated simulation. *Langauge and Cognition, 3*, 81 - 119. doi:10.1515/ LANGCOG.2011.004

Shinzato, K., & Torisawa, K. (2004). Acquiring hyponymy relations from web documents. In R. J. Mooney (Ed.), *Proceedings of Human Language Technology Conference/North American Chapter of the Association for Computational Linguistics Annual Meeting* (pp. 73-80). Stroudsburg, PA: Association for Computational Linguistics.

Shwartz, V., Goldberg, Y., & Dagan, I. (2016). Improving hypernymy detection with an integrated path-based and distributional model. *arXiv:1603.06076v3 [cs.CL]*.

Snow, R., Jurafsky, D., & Ng, A. Y. (2005). Learning syntactic patterns for automatic hypernym discovery. In L. Saul, Y. Weiss, & L. Bottou (Eds.), *Advances in Neural Information Processing Systems* (pp. 1297-1304). Cambridge, MA: MIT Press.

Weeds, J., Clarke, D., Reffin, J., Weir, D., & Keller, B. (2014). Learning to distinguish hypernyms and co-hyponyms. *Proceedings of COLING 2014, the 25th International Conference on Computational Linguistics: Technical Papers*, 2249-2259.

Wu, L.-L., & Barasalou, L. W. (2009). Perceptual simulation in conceptual combination: Evidence from property generation. *Acta Psychologica, 132*, 173-189.

Appendix 1: False Lists Used in Experiment 1

Anomalous False Items					
List 1			List 2		
Subject 1	Subject 2	Predicate	Subject 1	Subject 2	Predicate
DACQUIRI	GRADUATION	GRAIN	NOVEMBER	HIGHBALL	TIMPIECE
AUSTRALIA	CABINET	BEVERAGE	SYMPHONY	INCH	MEDICINE
ANT	CREEK	EXCREMENT	CALCULATOR	STOMACH	CRAFTSMAN
ENGLISH	OIL	TIME	JUPITER	PUSHUP	CONTAINER
CROW	NYLON	DRUG	GOAT	INDIANAPOLIS	UNDERWEAR
BOMB	IGLOO	REPTILE	SERGEANT	DAY	JEWELRY
JUICE	DICTIONARY	SHELLFISH	HALLOWEEN	COUSIN	DISTANCE
CHICAGO	LABOR-DAY	VEGETABLE	SWIMMING	NAPKIN	RODENT
SPRUCE	APPLE	FABRIC	GIRL	ELK	SCIENCE
CONVERTIBLE	PALM	SPICE	GURGLE	STAPLE	VEHICLE
TEMPERATURE	DACHSHUND	SEAFOOD	VOLCANO	CIDER	DWELLING
FUNERAL	SACK	GEM	COLT	ARKANSAS	GARDEN-TOOL
BLANKET	TELEPHONE	NUT	DEER	ROWBOAT	POLITICIAN
FRYING-PAN	AMPHETAMINE	STRING-INSTRUMENT	CALIFORNIA	COUNCILMAN	SILVERWARE
LIPSTICK	SOUR	TOY	ROOF	SYMPHONY	POLITICAL-SYSTEM
COAL	GATORADE	RELATIVE	WATER	BUBBLE-BATH	ROOM
ICED-TEA	VALLEY	TOOL	PHARMACIST	ARGENTINA	FISH
HARVESTER	WALTZ	MEAT	ELEPHANT	MUSTARD	BOOK
HATRED	LABORER	SHIP	CHESS	WASP	FUEL
LOLLIPOP	SLIP	WEAPON	LUNCH	BROOM	INSECT

Appendix 1: False Lists Used in Experiment 1 (cont.)

Coordinate False Items

List 1			List 2		
Subject 1	Subject 2	Predicate	Subject 1	Subject 2	Predicate
COKE	ROOT-BEER	PEPSI	VISION	TASTE	SMELL
TEA	MILK	COFFEE	CAN-OPENER	DISHWASHER	TOASTER
CAPTAIN	COLONEL	MAJOR	SMALLPOX	MUMPS	MEASLES
LIZARD	ALLIGATOR	CROCODILE	BANKER	GROCER	DRUGGIST
SHEEP	COW	HORSE	DUKE	QUEEN	KING
LUTHERAN	CATHOLIC	METHODIST	BOOT	SLIPPER	SANDAL
TAPE-RECORDER	RADIO	PHONOGRAPH	T-SHIRT	GIRDLE	BRA
BED	TABLE	CHAIR	BASEBALL	SOCCER	HOCKEY
TWEED	FLANNEL	CORDUROY	FILIPINO	CAMBODIAN	VIETNAMESE
WINE	VODKA	GIN	SHAMPOO	TOOTHBRUSH	TOOTHPASTE
BICYCLE	WAGON	TRICYCLE	SINCERITY	HONESTY	GENEROSITY
ANTELOPE	GIRAFFE	ZEBRA	THANKSGIVING	EASTER	CHRISTMAS
EARTHQUAKE	TORNADO	HURRICANE	CHIPMUNK	RABBIT	GROUNDHOG
STAPLE	GLUE	TAPE	KNIFE	SPOON	FORK
ELM	MAPLE	OAK	GOOSE	DUCK	TURKEY
RUBY	SILVER	GOLD	SOUTH-AMERICA	AFRICA	ASIA
SWEDE	FRENCHMAN	GERMAN	SECOND	HOUR	MINUTE
PENNY	NICKEL	DIME	CARDINAL	SPARROW	ROBIN
HYDROGEN	NITROGEN	OXYGEN	PEANUT	ALMOND	PECAN
BAG	BASKET	BOTTLE	TUBA	TRUMPET	TROMBONE

Appendix 1: False Lists Used in Experiment 1 (cont.)

Cross-Category False Items					
List 1			List 2		
Subject 1	Subject 2	Predicate	Subject 1	Subject 2	Predicate
ERASER	PAPER	WRITING-INSTRUMENT	MILE	KILOMETER	TIME
HOUR	MINUTE	DISTANCE	SNAIL	CAVIAR	SHELLFISH
WATERMELON	OLIVE	VEGETABLE	RICE	BREAD	PASTA
LIE	SEDUCTION	CRIME	CARNATION	LILY	TREE
RUSSIA	CHINA	CONTINENT	TROUSERS	MITTEN	FOOTWEAR
CLOSET	PATIO	ROOM	HAY	ALFALFA	GRAIN
SQUASH	RADISH	FRUIT	GUITAR	BANJO	WIND-INSTRUMENT
PAPER	WOOD	FABRIC	KEYS	GEARS	TOOL
LEG	ARM	ORGAN	CEILING	BASEMENT	BUILDING
LOBSTER	CLAM	FISH	WEASEL	FROG	REPTILE
CHURCH	SCHOOL	DWELLING	YUGOSLOVIAN	ROMAINIAN	ASIAN
CEDAR	MAPLE	FLOWER	HORSERADISH	VINEGAR	SPICE
SALAMANDER	BACTERIA	INSECT	SWEAT	SALIVA	EXCREMENT
OAT	BARLEY	NUT	URANIUM	CHROMIUM	GEM
ORANGE-JUICE	COFFEEMATE	DAIRY-PRODUCT	PLOW	WHEEL	VEHICLE
FIANCE	GUARDIAN	RELATIVE	HORNET	BUTTERFLY	BIRD
NECK	WAIST	JOINT	PERFUME	MOUTHWASH	COSMETIC
STANZA	VERSE	SONG	ASTROLOGY	HISTORY	SCIENCE
PEN	PENCIL	READING-MATERIAL	SCISSORS	WRENCH	WEAPON
HEROIN	NOVOCAINE	MEDICINE	SPATULA	PARING-KNIFE	SILVERWARE

Appendix 1: False Lists Used in Experiment 1 (cont.)

Reversed False Items

List 1			List 2		
Subject 1	Subject 2	Predicate	Subject 1	Subject 2	Predicate
TIMEPIECE	JEWELRY	WRISTWATCH	BOOK	READING-MATERIAL	DICTIONARY
APPLIANCE	MACHINE	WASHER	JOINT	BODY-PART	ELBOW
HERBIVORE	PREY	RABBIT	CARNIVORE	PREDATOR	TIGER
GUN	WEAPON	RIFLE	VEHICLE	SHIP	OCEAN-LINER
VEHICLE	AIRCRAFT	JET	ROOM	BUILDING-PART	KITCHEN
BIRD	PET	CANARY	GAME	SPORT	SOFTBALL
CHEESE	DAIRY-PRODUCT	MOZARELLA	OFFICE-SUPPLY	WRITING-INSTRUMENT	PEN
MEAT	POULTRY	TURKEY	METAL	FUEL	URANIUM
CURRENCY	MONEY	LIRA			
RODENT	PEST	MOUSE	STATE	CITY	NEW-YORK
REFRESHMENT	SNACK	JUICE	WOOD	TREE	ELM
MERCHANT	OCCUPATION	PHARMACIST	CANDY	SNACK	CHOCOLATE-BAR
ALCOHOL	COCKTAIL	SCOTCH	DISASTER	STORM	CYCLONE
SNAKE	REPTILE	PYTHON	NOISE	SOUND	GURGLE
RELATIVE	PARENT	FATHER	METAL	ELEMENT	IRON
SILVERWARE	KITCHEN-UTENSIL	KNIFE	FURNITURE	LIGHT-SOURCE	LAMP
MAMMAL	CARNIVORE	BEAR	FOOD	PASTA	SPAGHETTI
DISEASE	ILLNESS	CANCER	ROCK	FUEL	COAL
LIQUID	BEVERAGE	WATER	CROP	GRAIN	CORN
LANGUAGE	ASIAN	JAPANESE	LINEN	BEDDING	PILLOWCASE

Appendix 2

Similar and Dissimilar Coordinates Used in Experiment 2				
List 1				
Similar Coordinates			Dissimilar Coordinates	
Subject	Predicate		Subject	Predicate
television	stereo		television	dryer
washer	dryer		washer	stereo
robin	sparrow		robin	hawk
eagle	hawk		eagle	sparrow
encyclopedia	dictionary		encyclopedia	newspaper
magazine	newspaper		magazine	dictionary
cancer	heart-disease		cancer	mumps
measles	mumps		measles	heart-disease
nail	screw		nail	staple
paperclip	staple		paperclip	screw
chess	checkers		chess	roulette
poker	roulette		poker	checkers
bee	wasp		bee	ant
termite	ant		termite	wasp
pork	beef		pork	turkey
chicken	turkey		chicken	beef
sergeant	corporal		sergeant	colonel
general	colonel		general	corporal
duck	goose		duck	quail
pheasant	quail		pheasant	goose

Appendix 2 (cont.)

Similar and Dissimilar Coordinates Used in Experiment 2				
List 2				
Similar Coordinates			Dissimilar Coordinates	
Subject	Predicate		Subject	Predicate
chimpanzee	gorilla		chimpanzee	woman
man	woman		man	gorilla
Lutheran	Methodist		Lutheran	Hindu
Buddhist	Hindu		Buddhist	Methodist
diamond	sapphire		diamond	limestone
granite	limestone		granite	sapphire
physics	chemistry		physics	psychology
sociology	psychology		sociology	chemistry
salmon	tuna		salmon	anchovy
sardine	anchovy		sardine	tuna
salt	pepper		salt	ketchup
mustard	ketchup		mustard	pepper
soccer	hockey		soccer	volleyball
badminton	volleyball		badminton	hockey
soap	deodorant		soap	toothbrush
toothpaste	toothbrush		toothpaste	deodorant
pliers	wrench		pliers	saw
hammer	saw		hammer	wrench
pea	bean		pea	cucumber
squash	cucumber		squash	bean

Appendix 2 (cont.)

Similar and Dissimilar Coordinates Used in Experiment 2				
List 3				
Similar Coordinates			Dissimilar Coordinates	
Subject	Predicate		Subject	Predicate
sheet	blanket		sheet	boxspring
mattress	boxspring		mattress	blanket
coffee	tea		coffee	coke
pepsi	coke		pepsi	tea
Toyota	Datsun		Toyota	Mercedes
Cadillac	Mercedes		Cadillac	Datsun
lion	tiger		lion	wolf
fox	wolf		fox	tiger
trout	perch		trout	shark
barracuda	shark		barracuda	perch
apple	orange		apple	date
fig	date		fig	orange
bowl	cup		bowl	spoon
fork	spoon		fork	cup
sheet	pillowcase		sheet	towel
napkin	towel		napkin	pillowcase
guitar	banjo		guitar	trombone
trumpet	trombone		trumpet	banjo
piano	organ		piano	saxophone
clarinet	saxophone		clarinet	organ

Appendix 2 (cont.)

Similar and Dissimilar Coordinates Used in Experiment 2				
List 4				
Similar Coordinates			Dissimilar Coordinates	
Subject	Predicate		Subject	Predicate
aunt	uncle		aunt	father
mother	father		mother	uncle
emerald	ruby		emerald	silver
gold	silver		gold	ruby
mouse	rat		mouse	chipmunk
squirrel	chipmunk		squirrel	rat
king	queen		king	princess
prince	princess		prince	queen
tanker	ocean-liner		tanker	motorboat
rowboat	motorboat		rowboat	ocean-liner
pretzel	potato-chip		pretzel	cake
pie	cake		pie	potato-chip
hurricane	tornado		hurricane	snow
rain	snow		rain	tornado
clock	watch		clock	sundial
hourglass	sundial		hourglass	watch
jacks	marbles		jacks	erector-set
blocks	erector-set		blocks	marbles
bra	panties		bra	sweatshirt
T-shirt	sweatshirt		T-shirt	panties
